# An ECG–PPG Physiological Signal Emulator for Calibration and Validation of Cardiovascular Monitoring Devices

**DOI:** 10.3390/s26175578

**Published:** 2026-09-02

**Authors:** Thanh Ven Huynh, Trung Nghia Tran, Anh Tu Tran

**Affiliations:** 1 Research Laboratory, Faculty of Applied Science, Ho Chi Minh City University of Technology (HCMUT), 268 Ly Thuong Kiet Street, Dien Hong Ward, Ho Chi Minh City 72409, Vietnam; ven.huynh252@hcmut.edu.vn (T.V.H.); ttnghia@hcmut.edu.vn (T.N.T.); 2 Vietnam National University Ho Chi Minh City, Linh Xuan Ward, Ho Chi Minh City 71308, Vietnam

**Keywords:** ECG–PPG simulator, physiological signal emulator, Gaussian-based ECG model, multi-Gaussian PPG model, cardiovascular monitoring devices

## Abstract

Physiological signal emulators support the calibration, validation, and stress testing of cardiovascular monitoring devices. However, many existing systems generate electrocardiography (ECG) or photoplethysmography (PPG) independently and offer limited control over arrhythmia detection and ECG–PPG coupling. This study presents a programmable physiological signal emulator that integrates a unified event-driven ECG–PPG model with synchronized multichannel hardware. The model represents atrial pacing, atrioventricular conduction, and ventricular activation as separate functional blocks, enabling normal sinus rhythm, first-degree atrioventricular block, second-degree atrioventricular block Mobitz I, complete atrioventricular block, atrial tachycardia, and ventricular tachycardia. A Gaussian-based ECG is generated from the atrial and ventricular event sequences, while a multi-Gaussian PPG waveform is derived from ventricular activation using a beat-class-dependent electromechanical delay. The same processing architecture supports playback of recorded 12-lead clinical ECG data through an inverse lead transformation. The hardware uses an STM32F407VET6 microcontroller and MCP4921 digital-to-analog converters (DACs) to generate 10 synchronized analog outputs, comprising 09 ECG electrodes and 01 PPG channel. Validation covered physiological timing, analog-chain performance, and end-to-end signal reproduction. PR interval errors relative to a commercial electrocardiograph were 1.23 ms for normal sinus rhythm and 1.66 ms for first-degree atrioventricular block. The measured beat-to-beat PR increment during Mobitz I conduction was 40.02±0.04 ms for a programmed value of 40 ms. At commanded amplitudes of at least 800 mV, both output channels achieved absolute amplitude errors below 0.60%, total harmonic distortion below 1%, and signal-to-noise ratios (SNRs) above 30 dB. Inter-channel R-peak skew remained below the 2 ms sampling interval, and all monitored metrics varied by less than 1.5% during 60 min of continuous operation. Reproduction of a clinical 12-lead recording yielded per-lead $R^{2}$ values of 0.967–0.986 and a cycle-to-cycle correlation of 0.996. The emulator also reproduced amplitude-dependent bias in automated interval measurements and interpretation labels. These results demonstrate a low-cost, open-source platform for reproducible device calibration, algorithm stress testing, medical training, and physiological signal processing research.

## 1. Introduction

Cardiovascular disease is one of the leading causes of death worldwide [1]. According to the World Health Organization (WHO), approximately 19.8 million deaths were attributed to cardiovascular diseases in 2022, accounting for around 32% of all global deaths, with 85% of these due to myocardial infarction and stroke [2]. Against this backdrop, the development of early diagnostic techniques plays a critical role in mitigating premature cardiovascular mortality. Among these, electrocardiography (ECG) and photoplethysmography (PPG) are clinical tools used to record and assess the electrical activity of the heart and the peripheral hemodynamic response produced by the heart’s pumping action [3,4]. The combined use of ECG and PPG can provide a more comprehensive characterization of the cardiovascular system, capturing both cardiac electrical activity and peripheral vascular responses [5,6].

Numerous studies have focused on improving the accuracy of measuring devices, as well as signal-processing algorithms, to ensure the reliable acquisition, preservation, and analysis of signals [7,8,9]. In this context, physiological signal simulators have been developed as useful tools for testing, evaluating, and calibrating ECG devices, software, and biomedical signal-processing algorithms [10,11,12]. In addition to technical testing, simulation systems are also used in medical training, helping learners improve their skills in identifying and interpreting physiological signals, especially in complex pathological situations or cardiac arrhythmias [10,13,14]. Some studies have constructed traditional ECG simulators using oscillator circuits or oscillator-based integrated circuits to generate signal waveforms from simple periodic functions or discrete pulse sequences, rather than from full physiological models of the ECG [11]. Although the hardware structure is simple, easy to implement, and low-cost, it is limited in its ability to accurately reproduce the complex physiological variations of cardiac signals. Many studies have shifted toward an approach that uses real-world patient ECG data extracted from standard databases such as MIT-BIH or PhysioNet [13,15]. In this method, the ECG signal is processed, stored on a memory card, and then played back via a microcontroller combined with a digital-to-analog converter (DAC) to reconstruct the corresponding electrical signal [15]. This approach allows the simulation system to transmit data simultaneously across multiple signal channels and accurately reproduce the time variation between QRS complexes, thereby simulating heart rhythms that closely resemble clinical reality [13]. Despite its high fidelity, this method remains dependent on the input dataset, limiting scalability and the ability to generate new signal patterns beyond those already recorded. To reduce reliance on large storage requirements, another research direction is to use differential equations and mathematical functions to simulate cardiac dynamics [10]. Mathematical models used for physiological signal simulation include heterogeneous nonlinear oscillators, spatially discretized reaction–diffusion models, rings of three coupled oscillators, Gaussian wave-based motion models, and Gaussian wave-based state-space motion models [16,17,18]. However, model complexity and computational cost remain major challenges for hardware implementation. In addition, simulators that emulate ECG and PPG simultaneously are still limited, as most systems focus on either ECG or PPG [16,19,20]. The existing multimodal simulators provide limited independent control over signal amplitude, waveform morphology, temporal parameters, and physiological coupling between cardiac and peripheral signals [16,20]. This limitation restricts their use as general-purpose physiological signal emulators for the calibration and validation of cardiovascular monitoring devices.

To address this limitation, this study focuses on developing a programmable ECG–PPG physiological signal simulator that combines a unified mathematical model for ECG and PPG, synchronized with a multichannel hardware implementation, for calibrating and validating cardiovascular monitoring devices. The model in this study inherits the principles of ECG morphology representation using Gaussian functions and RR rhythm sequences from the electrocardiogram synthesizer (ECGSYN) [21], combined with a multilead vector model [22] and a PPG model based on multiple Gaussian functions [23]. However, the model does not directly use the phase-dependent dynamic equations of ECGSYN but is instead reformulated within an event-driven time-domain framework. In a phase-driven formulation, the P wave and QRS complex are generated within the same cardiac-cycle trajectory, limiting the independent representation of atrial impulses that are delayed or blocked before ventricular activation. By separating atrial and ventricular event sequences, the proposed formulation enables explicit modeling of atrioventricular conduction abnormalities. In this model, atrial activity, atrioventricular conduction, and ventricular activity are described by separate blocks in sequence: atrial pacemaker → conduction block → ventricular pacemaker → PPG generation block. This construction allows for the simulation of both normal sinus rhythm and abnormal rhythms involving altered atrial pacing, atrioventricular conduction, or ventricular activation. Simultaneously, the ventricular trigger sequence and RR interval are used as time inputs to the PPG block, thereby ensuring correlation between cardiac electrical activity and peripheral vascular response. The electromechanical delay and pulse amplitude further depend on beat class, so that an ectopic ventricular beat produces both a wide QRS complex and a delayed, attenuated peripheral pulse from a single event schedule. As a result, the simulated signal is formed from the pacing and conduction mechanisms, rather than simply binding P, Q, R, S, and T waves around a single R-peak sequence. The proposed framework also supports playback of clinical 12-lead ECG recordings via the same signal-generation pathway. An inverse lead transformation reconstructs the corresponding electrode-domain potentials from the recorded leads, after which both model-generated and database-derived ECG signals undergo the same downstream processing pathway. By integrating these two input modes within a unified hardware architecture, the system combines the configurability and scalability of mathematical synthesis with the morphological realism of clinical recordings, thereby addressing a major limitation of previous simulators [13,15,16]. This common processing framework further enables evaluation of hardware reproduction fidelity using clinically acquired waveforms, rather than exclusively against synthetic reference signals. The resulting system is evaluated on three levels: physiological plausibility against clinical ECG databases, reproduction fidelity and stability at the analog output, and signal acceptance by a commercial electrocardiograph. The main contributions of this paper are:

- Propose a coupled ECG–PPG model in which the peripheral pulse is generated from the ventricular activation sequence through a beat-class-dependent electromechanical delay, linking Gaussian-based ECG and multi-Gaussian PPG within a single event-driven structure;
- Design a block-based architecture that independently represents atrial pacing, atrioventricular conduction, and ventricular activation, enabling conduction patterns that are not directly supported by the standard single-cycle ECGSYN formulation;
- Integrate a clinical playback path that reconstructs electrode potentials from recorded 12-lead ECG data through an inverse lead transform, enabling model-generated and clinically recorded signals to be emulated by the same hardware;
- Implement a multichannel hardware platform that outputs synchronized analog ECG–PPG signals for connection to ECG recorders and to analog PPG acquisition or measurement interfaces;
- Validate the emulator against clinical recordings and at the analog output, quantifying morphological agreement, ECG–PPG timing preservation, and output stability across the complete digital-to-analog path.

## 2. Materials and Methods

### 2.1. Mathematical Model

The proposed model combines established representations of ECG morphology, multilead projection, and peripheral pulse morphology with an event-based framework for ECG–PPG synchronization. ECG wave components are represented using Gaussian functions derived from established synthetic ECG formulations [21], while multilead generation follows a three-dimensional (3D) cardiac vector approach [22]. The PPG waveform is represented by a reduced set of Gaussian components [23].

#### 2.1.1. Event Schedule and Atrioventricular Conduction

Atrial and ventricular activities are represented by separate event sequences. The event interval can include low-frequency modulation associated with respiratory sinus arrhythmia,

(1) $$t_{i+1}^{q}=t_{i}^{q}+\frac{60}{{\mathrm{HR}}_{q}}\left[1+\alpha_{q}\sin\left(2\pi f_{r}t_{i}^{q}\right)\right],\;q\in \left\{A,V\right\},$$

where ${\mathrm{HR}}_{q}$ denotes the mean event rate, $\alpha_{q}$ is the modulation depth, and $f_{r}$ is the modulation frequency. The modulation term follows the respiratory component used in ECGSYN [21], applied to the atrial and ventricular schedules rather than to a single cycle. Independent ventricular scheduling is used when ventricular activity is not driven by atrial conduction. A binary variable $c_{k}\in \left\{0,1\right\}$ defines conduction of the *k*-th atrial event, and atrioventricular conduction is represented on an event basis by

(2) $$t_{j}^{V}=t_{k}^{A}+{\mathrm{PR}}_{k},\;c_{k}=1,$$

where ${\mathrm{PR}}_{k}$ represents the atrioventricular conduction interval. Different conduction disorders are represented by the combination of the binary variable $c_{k}$ and the interval ${\mathrm{PR}}_{k}$. Normal conduction is defined by $c_{k}=1$ and ${\mathrm{PR}}_{k}={\mathrm{PR}}_{0}$, whereas first-degree block maintains 1:1 conduction with a prolonged ${\mathrm{PR}}_{k}$. Mobitz type I is represented by progressive prolongation of ${\mathrm{PR}}_{k}$ followed by a blocked atrial event with $c_{k}=0$. Complete atrioventricular block is modeled by setting $c_{k}=0$ for all atrial events, leaving ventricular activity governed by the independent ventricular schedule in Equation (1). The combination of ${\mathrm{PR}}_{k}$ and $c_{k}$ provides a compact representation of the principal atrioventricular conduction patterns for each atrial event. Separate atrial and ventricular schedules are maintained to accommodate both conducted and dissociated rhythms. The recovery-dependent prolongation underlying Mobitz type I reflects the dependence of atrioventricular nodal conduction time on the preceding recovery interval. Iterative application of the relationship across successive atrial events can reproduce conduction patterns represented by the pair $\left(c_{k},{\mathrm{PR}}_{k}\right)$ [24].

The modulation coefficients and baseline components are configurable. The present evaluation used $\alpha_{A}=\alpha_{V}=0$ and omitted baseline drift, so that any deviation observed at the analog output can be attributed to the signal chain rather than to model variability. The reference signals used consequently carry no heart-rate variability and no baseline wander.

#### 2.1.2. Multilead ECG Generation

A 3D cardiac source vector $v\left(t\right)={\left[X\left(t\right),Y\left(t\right),Z\left(t\right)\right]}^{T}$ represents the combined atrial and ventricular electrical activity, so that amplitude and timing relationships between leads remain mutually consistent by construction [22]. The P wave is associated with atrial events, whereas the Q, R, S, and T components are associated with ventricular events,

(3) $$v\left(t\right)=\sum_{k=1}^{N_{A}}G_{k}^{A}\;a_{P}\exp\;\left[-\frac{{\left(t-t_{k}^{A}-\mu_{P}\right)}^{2}}{2\sigma_{P}^{2}}\right]+\sum_{j=1}^{N_{V}}G_{j}^{V}\sum_{m\in \{Q,R,S,T\}}a_{m,c_{j}}\exp\;\left[-\frac{{\left(t-t_{j}^{V}-\mu_{m,c_{j}}\right)}^{2}}{2\sigma_{m,c_{j}}^{2}}\right],$$

(4) $$\phi_{e}\left(t\right)=p_{e}^{T}v\left(t\right)+b_{\mathrm{ECG}}\left(t\right)+n_{e}\left(t\right),$$

where a, μ, and σ define the amplitude vector, temporal position, and width of each component, the index $c_{j}$ distinguishes normal and ectopic ventricular beats. The coefficients $G_{k}^{A}$ and $G_{j}^{V}$ scale the atrial and ventricular contributions of the *k*-th and *j*-th events, comprising a global sensitivity setting and a beat-class factor that preserves the relative amplitude of ectopic beats. In Equation (4), $p_{e}$ defines the projection from the cardiac vector to electrode *e*, while $b_{\mathrm{ECG}}\left(t\right)$ and $n_{e}\left(t\right)$ denote the configurable baseline drift and additive noise terms, both set to zero in the present evaluation. The projection vectors are assigned so that the three limb electrodes sum to zero. The zero-sum constraint causes the Wilson central terminal to vanish for the synthesized source, allowing the precordial coordinates to be tuned without disturbing the limb leads, which would otherwise require iterative readjustment of all twelve leads whenever one precordial position was changed. The resulting electrode potentials are converted to the standard 12-lead ECG using the conventional Einthoven and Wilson relationships [25].

Normal and ectopic beats share the functional form of Equation (3) and differ only in the parameter set indexed by $c_{j}$. Ectopic activity is characterized by wider and later QRS components, eliminating the need for a separate generator. A global sensitivity coefficient scales the cardiac vector amplitude without altering event timing or wave duration. Depolarization duration is governed by tissue conduction velocity and is largely rate-independent, whereas repolarization follows the action potential duration and shortens as rate increases [26]. The model reproduces this asymmetry by scaling the T-wave temporal parameters,

(5) $$f_{T,j}=\mathrm{clip}\;\left[{\left(\frac{{\mathrm{RR}}_{j}}{{\mathrm{RR}}_{0}}\right)}^{\alpha_{\mathrm{QT}}},f_{\min},f_{\max}\right],\;\left\{\mu_{T},\sigma_{T}\right\}\to f_{T,j}\left\{\mu_{T},\sigma_{T}\right\},$$

where ${\mathrm{RR}}_{j}$ is obtained directly from the ventricular event sequence, ${\mathrm{RR}}_{0}$ is the reference interval, and $\alpha_{\mathrm{QT}}$ controls the rate dependence. The bounds $f_{\min}$ and $f_{\max}$ limit the scaling factor so that the T-wave position remains physically consistent at the extremes of the simulated rate range. Position and width are scaled by the same factor, so the repolarization segment is compressed uniformly and the T-wave shape is preserved. The direct inheritance of ${\mathrm{RR}}_{j}$ from the pacing and conduction blocks allows changes in rate or conduction pattern to propagate to repolarization automatically.

Recorded 12-lead ECG signals can enter the same electrode-domain pathway. The definition of ECG leads as potential differences limits the recovery of the electrode field to an arbitrary additive constant. Setting the right-arm potential as the reference resolves this ambiguity without affecting the reconstructed leads,

(6) $$\phi_{\mathrm{RA}}=0,\;\phi_{\mathrm{LA}}=I,\;\phi_{\mathrm{LL}}=\mathrm{II},\;\phi_{V_{i}}=V_{i}+\frac{I+\mathrm{II}}{3}.$$

The transformation reconstructs the nine electrode potentials required by the hardware while preserving the original lead relationships, enabling model-generated and recorded ECG data to share the same downstream signal-generation architecture. An alternative approach involves reconstructing an orthogonal source from the recorded leads and projecting the source through a regression-based lead transform [27]. This approach was not adopted due to the additional transformation error, which could reduce the fidelity of the reproduced recording.

#### 2.1.3. ECG–PPG Coupling and PPG Generation

The peripheral pulse is generated by ventricular events rather than by an independent timing sequence, since it follows mechanical ejection, and an atrial impulse that is delayed or blocked must produce no peripheral pulse at all. The pulse foot associated with the ventricular beat *j* is defined by

(7) $$t_{F,j}=t_{j}^{V}+{\mathrm{PAT}}_{j},\;{\mathrm{PAT}}_{j}={\mathrm{PEP}}_{j}+\frac{L}{{\mathrm{PWV}}_{j}},$$

where ${\mathrm{PEP}}_{j}$ denotes the pre-ejection period, *L* is the effective vascular path length, and ${\mathrm{PWV}}_{j}$ is the pulse wave velocity [28]. Equation (7) establishes the temporal coupling between the electrical and peripheral waveforms. The dependence on $t_{j}^{V}$ the derived conduction block enables conduction abnormalities represented by Equation (2) to propagate directly to the PPG waveform without additional specification. Contractility and arterial stiffness coefficients permit adjustment of PEP and PWV. Rate-dependent coefficients were set to zero during the present evaluation to isolate the prescribed electromechanical delay from any confounding rate effect.

Beat class also modifies peripheral pulse generation. Ventricular ectopic beats have a shorter diastolic filling time and a dyssynchronous contraction and are therefore assigned lower contractility and pulse amplitude than normal beats. Because the class index $c_{j}$ governs both Equation (3) and the peripheral parameters, one event schedule produces a wide QRS complex together with a delayed and attenuated peripheral pulse. Generating the two signals from separate models and aligning them afterward was rejected because consistency under arrhythmia would then have to be imposed externally for every rhythm.

Each peripheral pulse is represented by five Gaussian components [23],

(8) $$p_{j}\left(\tau \right)=A_{j}\;u\left(\tau \right)\sum_{g=1}^{5}a_{g}\exp\;\left[-\frac{{\left(\tau -\mu_{g}\right)}^{2}}{2\sigma_{g}^{2}}\right],\;\tau =t-t_{F,j},$$

where $A_{j}$ is the beat-dependent pulse amplitude and $a_{g}$, $\mu_{g}$, and $\sigma_{g}$ define the morphology of each component. The smooth onset function u(τ) suppresses the waveform before the pulse foot. Two modifications were introduced relative to the source formulation. The dicrotic notch is generated through the overlap of adjacent components along the descending phase rather than by an additional negative component. This representation avoids parameter settings that could produce nonphysiological negative amplitudes. The model also represents only the pulsatile component, consistent with the study focus on waveform morphology and temporal coupling rather than absolute optical intensity. A common ventricular event sequence coordinates ECG morphology and peripheral pulse generation. Changes in cardiac rhythm, conduction, or beat class consequently propagate through both signals without requiring independent post-generation alignment.

### 2.2. Overall System Flowchart

**Figure 1:**
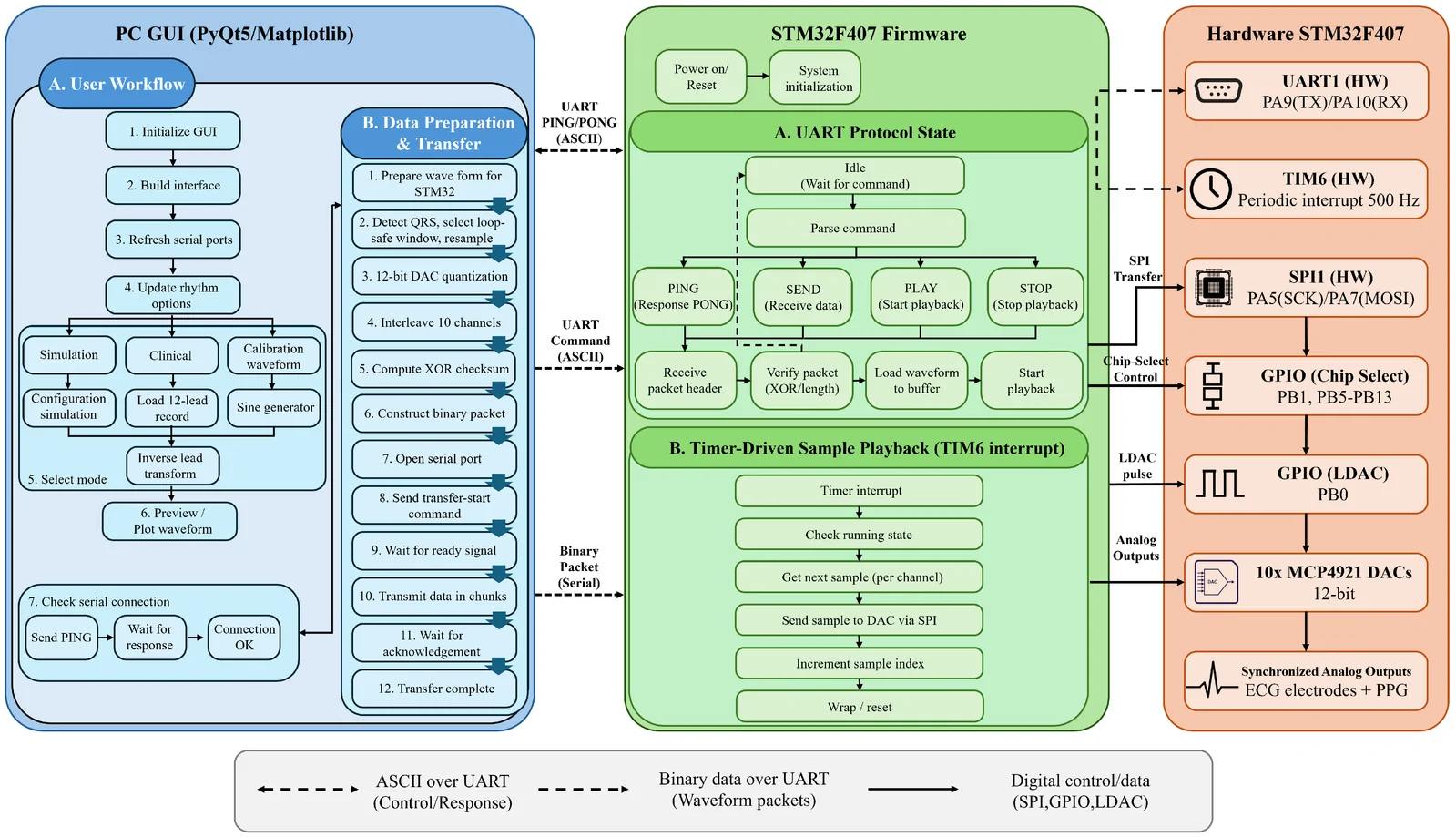
Architectural diagram and overall operation flowchart of a synchronized ECG–PPG signal generation system using an OEM STM32F407VET6-based development board (STM32F407VET6 microcontroller, STMicroelectronics, Geneva, Switzerland).

The overall system architecture describes the processing sequence from waveform configuration on the computer to synchronized analog signal generation by the hardware, as shown in Figure 1. The system comprises a software stage and an embedded hardware stage based on the STM32F407VET6. The software, developed using Python version 3.12.10, PyQt5 version 5.15.11, and Matplotlib version 3.10.9, performs parameter configuration, ECG–PPG synthesis, waveform visualization, data preparation, and transmission. Both model-generated signals from Section 2.1 and clinical 12-lead recordings converted to electrode potentials using Equation (6) enter the same processing pathway. The generated waveforms are mapped to the 12-bit DAC range according to

(9) $$D_{N}=\mathrm{round}\left[\frac{x+V_{\mathrm{FS}}}{2V_{\mathrm{FS}}}\left(2^{n}-1\right)\right],\;n=12,$$

where $V_{\mathrm{FS}}$ denotes the half-scale voltage range of each output channel. The ECG channels use $V_{\mathrm{FS},\mathrm{ECG}}=5.476\;\mathrm{mV}$ at the electrode terminals, corresponding to a quantization step of approximately 2.67μV. The PPG channel uses $V_{\mathrm{FS},\mathrm{PPG}}=1.25\;V$ without output attenuation, corresponding to a quantization step of approximately 0.61mV. The quantized samples are then organized for transmission to the microcontroller.

The STM32F407VET6 receives the waveform data through UART, verifies the transmitted packets, and stores valid samples in memory before playback. A TIM6 interrupt controls signal generation at $f_{s}=500\;\mathrm{Hz}$, corresponding to a sampling period of 2ms and a Nyquist frequency of 250Hz. At each sampling instant, the microcontroller transfers one sample from each channel to the ten MCP4921 DACs through SPI. A common LDAC pulse subsequently updates all converter outputs simultaneously, preventing inter-channel timing offsets associated with sequential SPI transmission. The resulting architecture separates waveform generation and parameter control from deterministic real-time signal output. A common sampling clock and simultaneous DAC update preserve temporal alignment across all ECG and PPG channels, including the relative delay between cardiac electrical activity and the peripheral pulse waveform. The selected sampling rate provides a Nyquist frequency of 250 Hz, which exceeds the 150 Hz upper bandwidth specified for diagnostic electrocardiography and matches the 500 Hz sampling rate of the reference recordings used for clinical playback [25]. Increasing the update rate to 1 kHz would not improve waveform fidelity without a corresponding increase in analog bandwidth, as the low-pass stage in Section 2.3.2 limits the reproduced spectrum to approximately 156 Hz. The 500 Hz rate was consequently selected as a system-level design trade-off rather than a hardware constraint, since transmission of all ten DAC command words occupies only about 1% of each sampling period. Applications involving high-frequency QRS analysis or ventricular late potentials would require both a higher update rate and a redesigned analog filter stage.

### 2.3. Hardware Implementation

**Figure 2:**
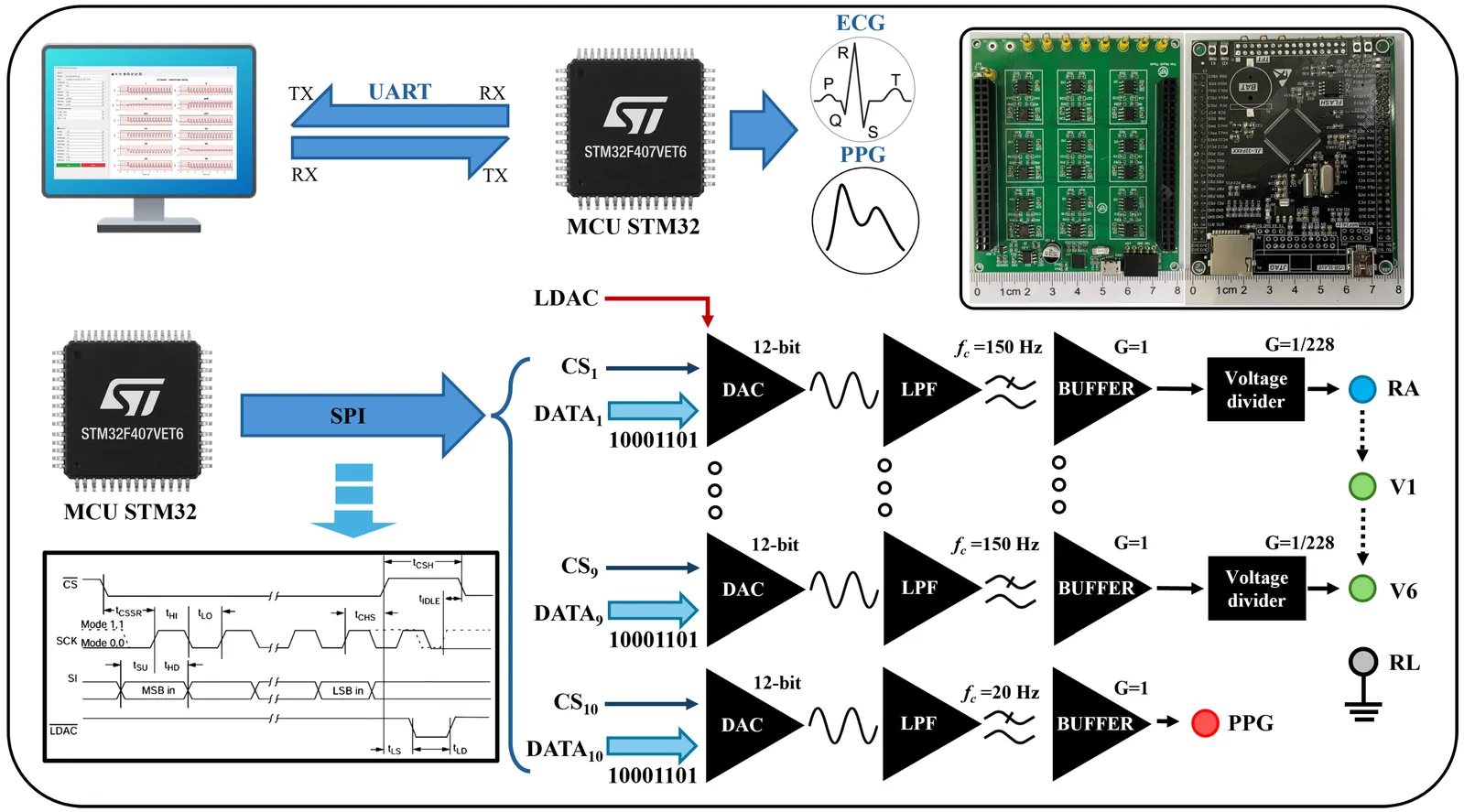
Block diagram of the electrophysiological signal simulation model.

The hardware model comprises a microcontroller, DACs, low-pass filters (LPFs), buffer stages, and voltage dividers, as shown in Figure 2. The STM32F407VET6 controls waveform playback at a sampling frequency of 500Hz using a TIM6 timer interrupt. At each sampling instant, ten digital samples are transferred to the MCP4921 DACs through SPI, followed by a common LDAC trigger for simultaneous output updating. This architecture maintains temporal synchronization between the ECG and PPG channels.

The DAC outputs are filtered to suppress conversion-related high-frequency components and are subsequently buffered to minimize loading effects. The ECG outputs are further attenuated by a 75kΩ/330Ω resistive divider, providing an attenuation ratio of approximately 1:228 and scaling the signals to the millivolt range required for ECG measurement. The resulting signal chain converts the synthesized digital waveforms into synchronized and appropriately scaled analog ECG and PPG outputs.

The complete system occupies a board area of 80 mm × 90 mm and draws approximately 145 mA during simultaneous operation of all ten channels. Power is supplied through the USB connection used for data transfer, eliminating the need for an additional power source. The measured current corresponds to approximately 29% of the 500 mA current limit of a USB 2.0 port. The shared power and communication interface also simplifies integration with external measurement equipment. The estimated component cost is approximately 45 USD, supporting implementation in teaching laboratories, prototyping environments, and routine quality-control applications that may require multiple units.

#### 2.3.1. Digital-to-Analog Converter Block

**Figure 3:**
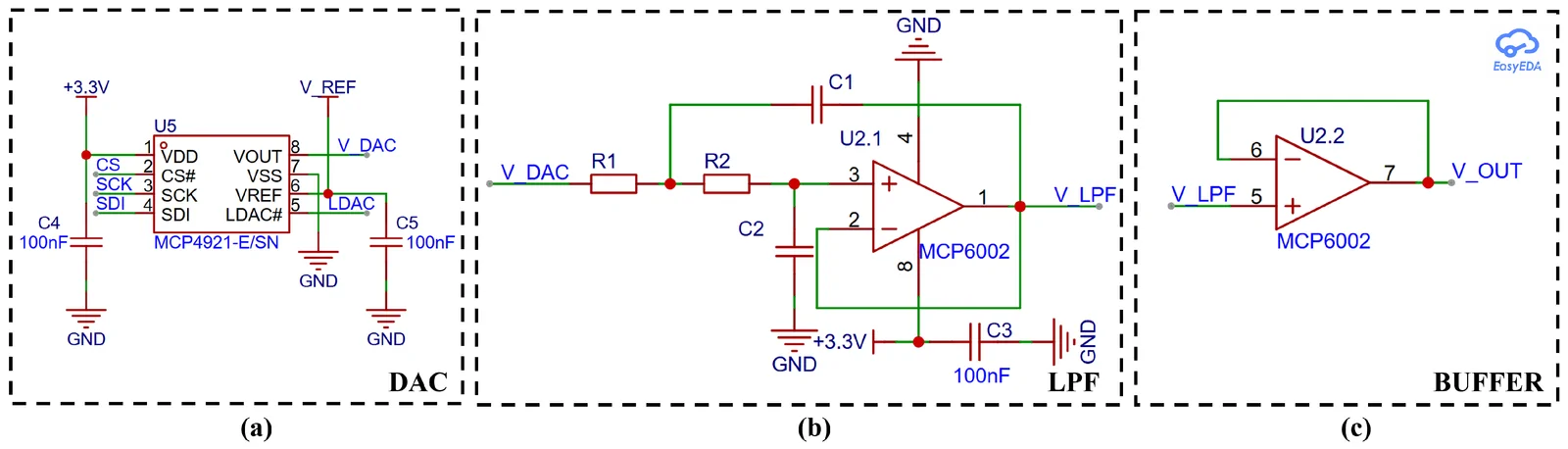
Schematic diagram of a signal channel: (**a**) DAC circuit, (**b**) LPF circuit, and (**c**) buffer circuit. Colors follow the default EasyEDA Pro schematic convention: green lines indicate electrical connections, red symbols represent circuit components and power/ground terminals, and blue text denotes signal/net and component labels. The schematic was drawn using EasyEDA Pro version 2.2.45.

The digital-to-analog conversion stage uses ten 12-bit MCP4921 DACs to reconstruct the synthesized ECG and PPG waveforms. Nine converters generate the ECG electrode potentials (RA, LA, LL, and V1–V6), while one converter generates the PPG waveform (Figure 3a). The 12-bit resolution provides 4096 discrete output levels, with the analog voltage determined by

(10) $$V_{\mathrm{DAC}}=\frac{V_{\mathrm{REF}}GD_{N}}{2^{n}},$$

where $D_{N}$ is the digital input code, *G* is the gain setting, $V_{\mathrm{REF}}=2.5\;V$ is the reference voltage, and n=12 is the resolution. The STM32F407VET6 transfers 16-bit command words to the DACs through SPI. Independent conversion of the nine ECG electrode potentials preserves the spatial voltage relationships generated by the 3D cardiac vector model. Synchronization across all ten channels is achieved through the external LDAC function of the MCP4921. The 16-bit command words are transferred sequentially through SPI and applied to the analog outputs by a common LDAC pulse, enabling simultaneous updates of the ECG and PPG channels. The 20 MHz SPI clock permits transmission of all ten command words within 20 μs. The total transfer and settling duration remains approximately 1% of the 2 ms sampling interval, based on the typical DAC settling time of 4.5 μs. The use of individual DACs increases component count and chip-select requirements, while providing deterministic multichannel synchronization. A multichannel DAC could reduce hardware complexity if equivalent simultaneous-update capability is maintained. The reconstructed analog signals are subsequently processed by the LPF and buffer stages described in Section 2.3.2 and Section 2.3.3.

#### 2.3.2. Low-Pass Filter Block

The LPF stage was incorporated to suppress high-frequency components introduced by digital-to-analog conversion and to improve waveform continuity at the system output. A second-order Sallen–Key configuration with unity gain ($A_{0}=1$) was adopted, as shown in Figure 3b. Equal resistor and capacitor values yield a quality factor of Q=0.5, corresponding to a critically damped response with a monotonic magnitude characteristic and no resonant amplification [29]. A Butterworth configuration (Q=0.707) provides a steeper transition band but introduces time-domain overshoot and ringing, which can distort the QRS morphology. Since amplitude fidelity and time-domain waveform integrity are the primary requirements for a calibration source, the critically damped configuration was preferred, accepting a slower roll-off and a larger baseline group delay in exchange for a non-overshooting response. The corresponding −3 dB cutoff frequency and low-frequency group delay are

(11) $$f_{-3\mathrm{dB}}=\frac{\sqrt{\sqrt{2}-1}}{2\pi RC}\approx \frac{0.644}{2\pi RC},\;\tau_{g}\left(0\right)=\frac{1}{Q\omega_{0}}=2RC.$$

The cutoff frequencies were defined according to the spectral characteristics of the reconstructed physiological signals. The ECG channel uses $C_{1}=C_{2}=100\;\mathrm{nF}$ and $R_{1}=R_{2}=6.8\;k\Omega$, corresponding to $f_{-3\mathrm{dB}}\approx 150.73\;\mathrm{Hz}$. The selected bandwidth preserves clinically relevant ECG components, including the higher-frequency content associated with the QRS complex [25]. The PPG channel uses the same capacitance with $R_{1}=R_{2}=51\;k\Omega$, giving $f_{-3\mathrm{dB}}\approx 20.10\;\mathrm{Hz}$. This cutoff is consistent with the predominantly low-frequency spectral content of the PPG waveform.

The difference in cutoff frequency also produces a deterministic difference in low-frequency group delay between the two channels. Equation (11) gives $\tau_{g}=1.36\;\mathrm{ms}$ for the ECG channel and 10.20ms for the PPG channel. The resulting differential delay is

(12) $$\Delta \tau =2C(R_{\mathrm{PPG}}-R_{\mathrm{ECG}})=8.84\;\mathrm{ms}.$$

The PPG waveform consequently exhibits an additional delay of approximately 8.84ms relative to the ECG waveform at low frequencies. Such a deterministic offset can introduce a systematic contribution to the reproduced pulse arrival time.

#### 2.3.3. Buffer Stage

The buffer stage isolates the LPF from the measurement load and preserves the reconstructed waveform at the system output. Each channel employs an MCP6002 operational amplifier configured as a unity-gain voltage follower (Figure 3c). The high input impedance limits loading of the Sallen–Key network, whereas the low output impedance reduces changes in filter response and signal amplitude caused by the 75.3kΩ resistive divider and the input impedance of the connected recorder.

The MCP6002 provides rail-to-rail input and output operation from a single supply and a gain–bandwidth product of 1MHz. The available bandwidth exceeds the 150Hz ECG cutoff by approximately $6.6\times 10^{3}$, corresponding to an estimated group delay of approximately 0.2μs. This delay is smaller than the differential delay caused by the LPF in (12), indicating that the buffer contributes negligibly to the inter-channel time difference. The accuracy and noise performance of the complete DAC, LPF, and buffer chain are evaluated experimentally in Section 3.2.

### 2.4. Evaluation Methods

The evaluation comprised four components: characterization of the synthesized signals, assessment of ECG–PPG electromechanical coupling, comparison with clinical data, and analysis of hardware accuracy and stability. Respiratory modulation and baseline drift were disabled during evaluation to provide deterministic reference signals and isolate deviations introduced by signal generation and hardware processing. The measured parameters were evaluated against the tolerance limits specified for diagnostic ECG in ANSI/AAMI EC11 [30] and IEC 60601-2-25 [31]. Although both standards address recording devices rather than signal sources, their limits provide an appropriate benchmark for assessing calibration-source performance. A calibration source should exhibit measurement errors substantially lower than the tolerances of the device under evaluation.

#### 2.4.1. Characterization of the Synthesized Signals

The synthesized signals were evaluated in terms of temporal parameters, multilead ECG morphology, and rhythm-dependent conduction patterns. Fiducial points were extracted from the P wave, QRS complex, T wave, and PPG pulse. The PPG foot was identified using the intersecting tangent method [32]. Deviations from prescribed parameters were quantified using the relative error

(13) $$E_{x\%}=\frac{|x_{\mathrm{meas}}-x_{\mathrm{set}}|}{|x_{\mathrm{set}}|}\times 100\%,$$

where $x_{\mathrm{set}}$ and $x_{\mathrm{meas}}$ denote the prescribed and measured values, respectively. The evaluated ECG parameters included heart rate, P-wave duration and amplitude, PR interval, QRS duration, QT interval, R-wave amplitude at V_5_, S-wave depth at V_1_, and mean electrical axis. Rhythm-dependent conduction was characterized using the PR interval, atrioventricular event ratio, atrial and ventricular rates, and QRS duration.

#### 2.4.2. Assessment of ECG–PPG Electromechanical Coupling

Electromechanical coupling was evaluated using pulse arrival time (PAT). The measured PAT was obtained from the interval between the ECG R peak and PPG pulse foot and compared with the prescribed value ${\mathrm{PAT}}_{\mathrm{set}}={\mathrm{PEP}}_{j}+L/{\mathrm{PWV}}_{j}$. The signed PAT error was defined as

(14) $$E_{\mathrm{PAT}}={\mathrm{PAT}}_{\mathrm{meas}}-{\mathrm{PAT}}_{\mathrm{set}}.$$

Digital consistency was assessed by comparing the measured PAT with the corresponding model value. Hardware preservation of ECG–PPG timing was evaluated by simultaneous oscilloscope measurement of the LL and PPG outputs. The difference between hardware and digital PAT quantified the timing contribution of the DAC, LPF, and buffer stages. Beat-dependent coupling was examined by comparing PAT and pulse amplitude between normally conducted and ventricular-origin beats. Group differences were evaluated using an independent two-sample *t*-test or Mann–Whitney test according to the data distribution, with p<0.05.

#### 2.4.3. Comparison with Clinical Data

The physiological plausibility of the synthesized waveforms was assessed against ECG recordings from PhysioNet [33]. Signals were resampled to a common sampling frequency, baseline corrected, temporally aligned, and amplitude normalized before comparison.

Waveform similarity was quantified using the Pearson correlation coefficient [34,35],

(15) $$r=\frac{\sum_{n=1}^{N}\left(x\left[n\right]-\bar{x}\right)\left(\hat{x}\left[n\right]-\bar{\hat{x}}\right)}{\sqrt{\sum_{n=1}^{N}{\left(x\left[n\right]-\bar{x}\right)}^{2}}\sqrt{\sum_{n=1}^{N}{\left(\hat{x}\left[n\right]-\bar{\hat{x}}\right)}^{2}}},$$

the percentage root-mean-square difference (PRD) [36,37],

(16) $$\mathrm{PRD}=\sqrt{\frac{\sum_{n=1}^{N}{\left(x\left[n\right]-\hat{x}\left[n\right]\right)}^{2}}{\sum_{n=1}^{N}x^{2}\left[n\right]}}\times 100\%,$$

and the root mean square error (RMSE) [34,35],

(17) $$\mathrm{RMSE}=\sqrt{\frac{1}{N}\sum_{n=1}^{N}{\left(x\left[n\right]-\hat{x}\left[n\right]\right)}^{2}}.$$

The ECG comparison also included PR interval, QRS duration, QT interval, QTc, R-wave amplitude at V_5_, and S-wave depth at V_1_.

#### 2.4.4. Hardware Accuracy and Stability

Hardware performance was evaluated across the DAC, LPF, and buffer stages. Sinusoidal signals generated by the STM32 were used to characterize the DAC, while the LPF and buffer were tested using a AFG3252C function generator (Tektronix, Inc., Beaverton, OR, USA). Output waveforms were acquired using a MDO3052 oscilloscope (Tektronix, Inc., Beaverton, OR, USA). Amplitude error was calculated from the relative difference between the prescribed and measured amplitudes.

Nonlinear distortion was evaluated using total harmonic distortion (THD) [38],

(18) $$\mathrm{THD}=\frac{\sqrt{\sum_{h=2}^{H}V_{h}^{2}}}{V_{1}}\times 100\%.$$

Baseline stability was assessed from the change in DC offset during 60 min of continuous operation. Signal quality was quantified using the signal-to-noise ratio (SNR) [38],

(19) $$\mathrm{SNR}=20\log_{10}\left(\frac{V_{\mathrm{sig},\mathrm{rms}}}{V_{\mathrm{noise},\mathrm{rms}}}\right).$$

Measurement repeatability was expressed using the coefficient of variation [39],

(20) $$CV_{p}=\frac{\sigma_{p}}{\bar{p}}\times 100\%,$$

where $\bar{p}$ and $\sigma_{p}$ denote the mean and standard deviation of the evaluated parameter. Amplitude, frequency, offset, and SNR were examined across repeated measurements to characterize the stability of the analog output chain.

## 3. Results

### 3.1. Physiological and Mathematical Validation of the Synthesized Signals

#### 3.1.1. Multilead Morphology and Lead Consistency

**Figure 4:**
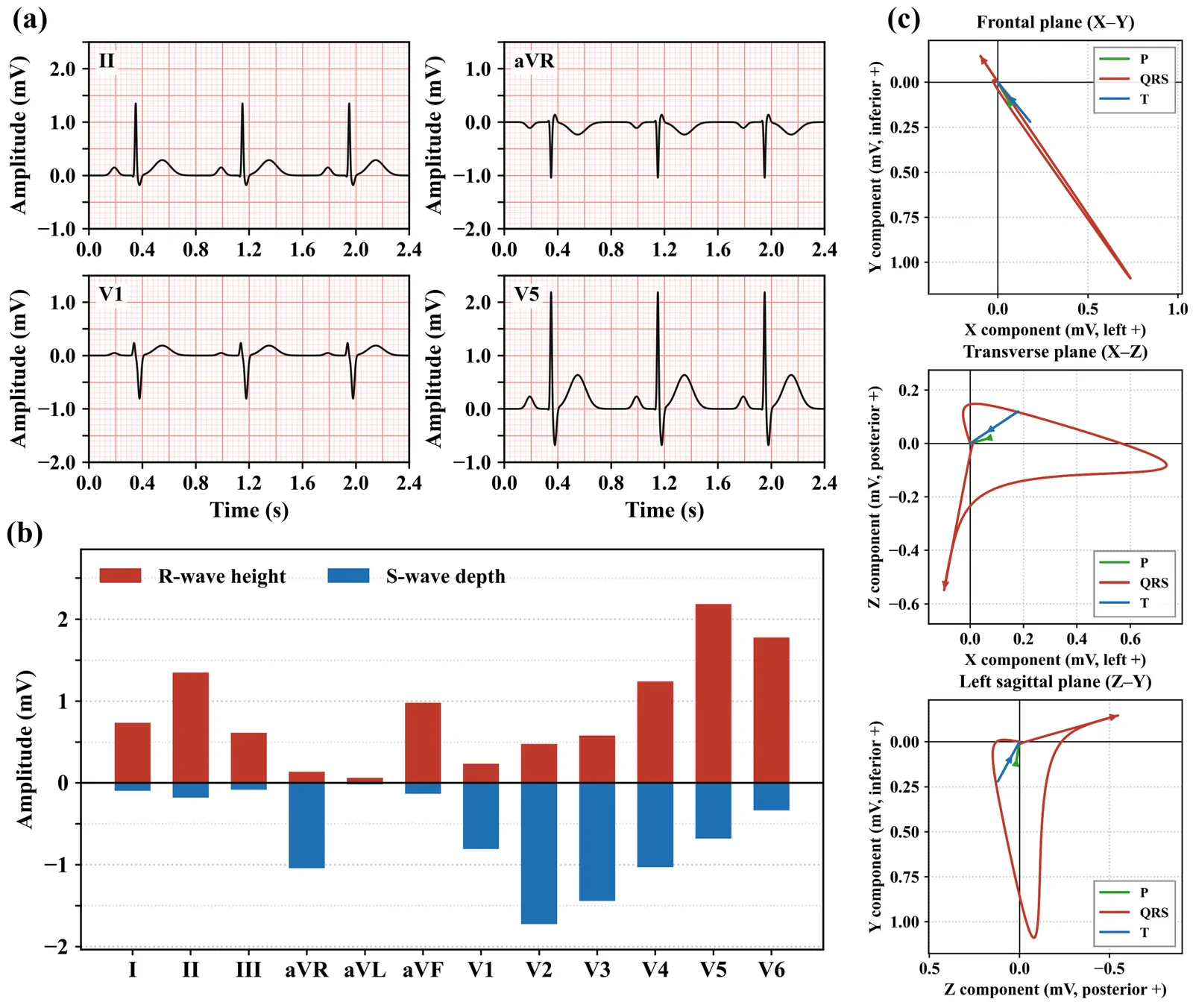
Morphological and vectorcardiographic characterization of the synthesized 12-lead ECG under normal sinus rhythm at 75 bpm and an amplitude scaling of 1 mV: (**a**) representative ECG waveforms from leads II, aVR, V1, and V5, illustrating rhythm, polarity, and precordial morphology, (**b**) R-wave height and S-wave depth across the standard 12 leads, and (**c**) vectorcardiographic loops of the P wave, QRS complex, and T wave projected onto the frontal, transverse, and left sagittal planes.

The multilead morphology and vectorcardiographic consistency of the synthesized ECG model were evaluated directly in the digital domain under normal sinus rhythm at 75 bpm and a nominal amplitude scaling of 1 mV. Figure 4 presents representative lead waveforms, the distribution of R-wave height and S-wave depth across the 12 standard leads, and the vectorcardiographic trajectories of the P wave, QRS complex, and T wave in three orthogonal planes.

Lead II exhibited a predominantly positive QRS complex with an R-wave amplitude of 1.348 mV, whereas lead aVR showed predominantly negative deflection with a minimum amplitude of −1.042 mV (Figure 4a). Across the precordial leads, the S wave reached the greatest depth in V2. The R-wave amplitude increased progressively and reached a maximum of 2.186 mV in V5 before decreasing in V6. The R/S ratio changed from less than 1 in V1–V3 to greater than 1 in V4–V6, indicating a precordial transition zone between V3 and V4 (Figure 4b). The mean frontal-plane axes of the P wave, QRS complex, and T wave were 60.3°, 56.9°, and 50.7°, respectively, indicating closely aligned vector orientations (Figure 4c). The residuals of the lead relationships (II−I−III) and (aVR+aVL+aVF) were on the order of $10^{-13}\;\mu V$, demonstrating that the algebraic relationships among the limb leads were preserved within numerical precision.

#### 3.1.2. Rhythm and Conduction Behavior

**Figure 5:**
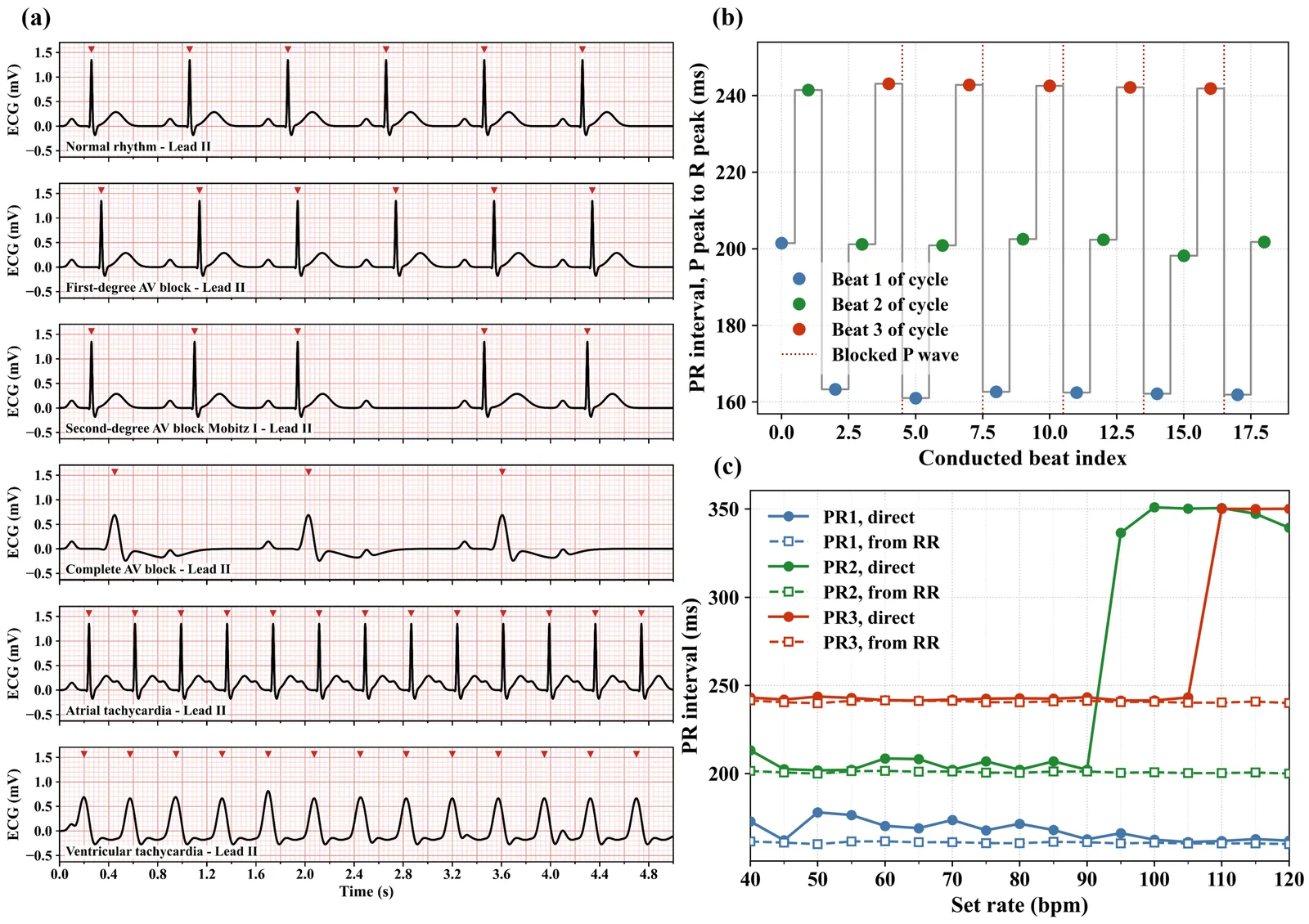
Conduction behavior of the block-based rhythm generator: (**a**) Lead II waveforms for the six implemented rhythms, with triangles marking QRS complexes, (**b**) progressive PR prolongation within each Mobitz I cycle at 75 bpm and 1 mV, comparing the prescribed values with those reported by the Kenz Cardico 306, and (**c**) agreement between direct and RR-derived PR measurements across the evaluated heart-rate range at 1 mV.

Conduction behavior was evaluated by comparing the prescribed event schedule with measurements obtained from the Kenz Cardico 306 electrocardiograph (Suzuken Co., Ltd., Nagoya, Aichi, Japan). The analysis focused on atrioventricular conduction and PR interval accuracy in Mobitz type I rhythm, while residual discrepancies were examined to distinguish recorder-related measurement effects from waveform-generation errors. Figure 5 presents the lead II waveforms of six rhythm types, the variation in PR interval according to the sequence of conducted beats in Mobitz I, and the agreement between direct measurements and PR values derived from the RR cycle.

Figure 5a shows that the algorithm reproduced the characteristic conduction patterns of each rhythm. Normal sinus rhythm, first-degree atrioventricular block, and atrial tachycardia maintained one P wave for each QRS complex. In the analyzed Mobitz I segment, six P waves were accompanied by five QRS complexes because one atrial impulse was not conducted. The complete atrioventricular block segment contained six P waves and three QRS complexes, reflecting atrioventricular dissociation. Ventricular tachycardia exhibited more QRS complexes than P waves, consistent with a predominantly ventricular rhythm source. For Mobitz I, the programmed PR interval increased sequentially from 160 to 240 ms before the dropped beat. Direct measurements from the Kenz Cardico 306 yielded corresponding mean values of 166.74±15.97, 270.62±71.62, and 271.40±48.01 ms, with a pooled increment of 46.92±22.56 ms compared with the programmed increment of 40 ms (Figure 5b). In contrast, the PR intervals derived from the RR cycle more closely matched the programmed values, with an increment of 40.00±0.11 ms and mean errors of approximately 0.78–0.79 ms across the three PR positions. The comparison in Figure 5c indicates that the discrepancy primarily arose from the recorder’s direct PR interval delineation, particularly at higher heart rates, rather than from the temporal structure generated by the algorithm.

The conduction parameters measured by the Kenz Cardico 306 generally agreed with the model settings (Table 1). The PR interval errors were 1.23 ms for NSR and 1.66 ms for 1° AVB. In Mobitz I, the measured PR interval increased from 161.13±0.32 to 241.16±0.25 ms, while the beat-to-beat PR increment was 40.02±0.04 ms compared with the prescribed value of 40 ms. For CAVB, the measured ventricular rate was 38.02±0.00 bpm, close to the prescribed rate of 38 bpm. Atrial and ventricular tachycardia reached 159.59±0.00 and 159.57±0.00 bpm, respectively, while maintaining QRS durations consistent with the predefined criteria. These results confirm that the system reproduced the characteristic conduction and rate parameters of each rhythm.

#### 3.1.3. ECG–PPG Coupling

**Figure 6:**
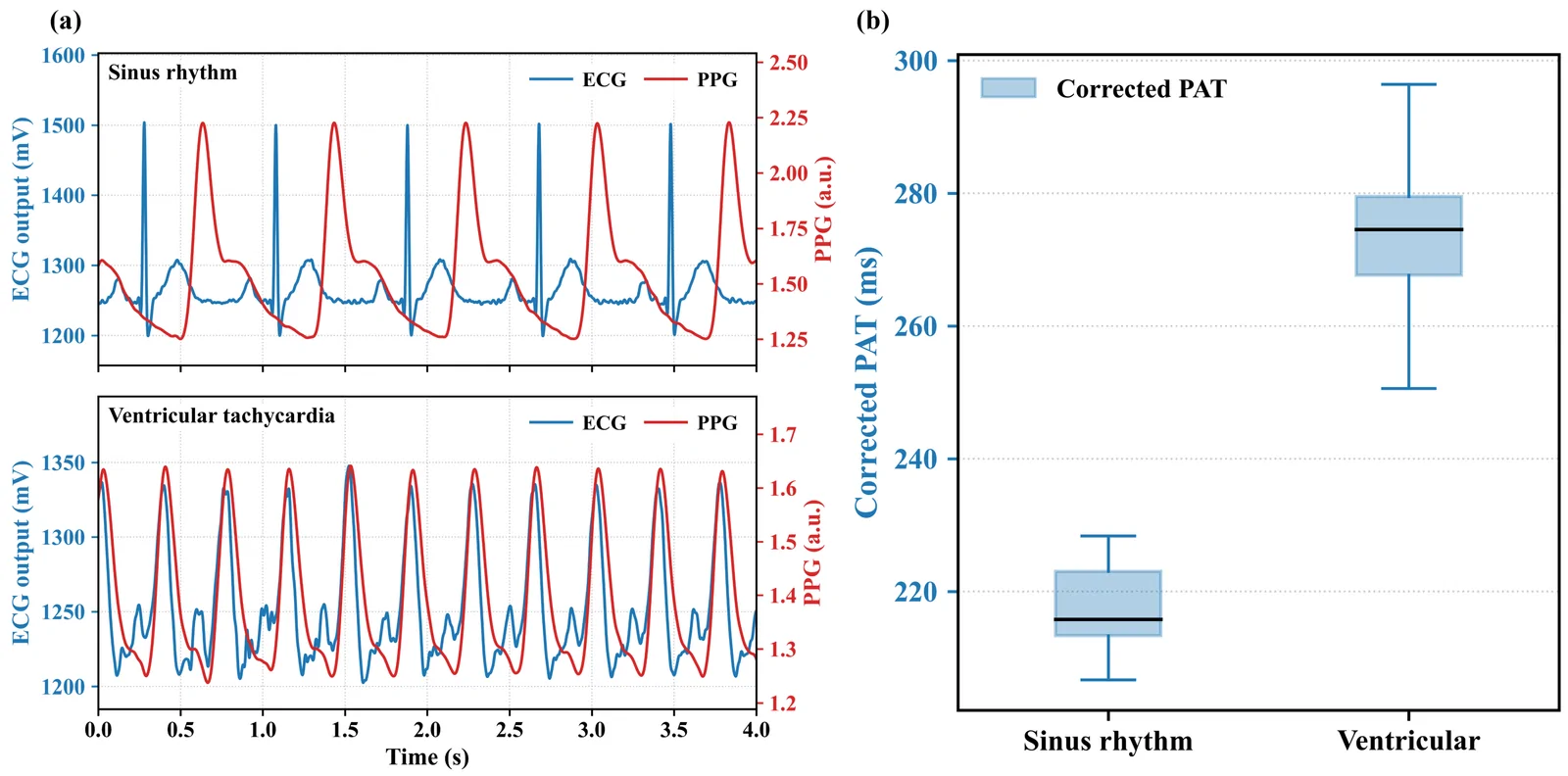
Rhythm-dependent electro-mechano-vascular coupling: (**a**) simultaneous ECG and PPG waveforms acquired on two oscilloscope channels at the analog buffer outputs, for sinus rhythm at 75 bpm and 2 mV (**top**), and ventricular tachycardia at 140 bpm and 2 mV (**bottom**); (**b**) beat-level distribution of the PAT for the two rhythms.

Beat-dependent modulation of peripheral pulse timing and amplitude was evaluated from the simultaneously measured ECG and PPG outputs. Figure 6 characterizes the resulting rhythm-dependent electromechanical coupling using the analog waveforms and corresponding pulse arrival time measurements. In sinus rhythm, each R-peak was consistently associated with a corresponding PPG pulse foot, yielding a mean PAT of 217.48 ms compared with the prescribed value of 209.09 ms, an error of 8.39 ms, and an amplitude ratio of 1.00. In contrast, ventricular tachycardia produced shorter cardiac cycles, increased PAT to 273.92 ms compared with the prescribed value of 244.09 ms, and reduced the amplitude ratio to 0.44 (Table 2). The distributions in Figure 6b show clear separation between the two PAT groups, with ventricular tachycardia exhibiting an analog-output delay approximately 56 ms greater than that of sinus rhythm.

The PAT error was 29.83 ms in ventricular tachycardia compared with 8.39 ms in sinus rhythm. The increase accompanied a reduction in the amplitude ratio to 0.44, which lowers the reliability of pulse-foot detection given the reduced signal amplitude and altered upstroke morphology. Simultaneous ECG and PPG generation enables controlled reproduction of electro-mechano-vascular changes, including pre-ejection time, pulse amplitude, and vascular delay, which cannot be represented by an ECG-only model.

### 3.2. Analog Output Fidelity, Synchronization, and Stability

#### 3.2.1. Analog-Chain Accuracy

The accuracy of the analog signal chain was evaluated in terms of frequency response, amplitude error, total harmonic distortion, and signal-to-noise ratio. The experimental magnitude and phase responses were compared with the corresponding simulation results for the ECG and PPG channels. Amplitude error, THD, and SNR were examined over a commanded voltage range of 50–1200 mV using 10 Hz and 5 Hz sinusoidal signals for the ECG and PPG channels, respectively. The errors introduced by the DAC, LPF, and output buffer stages were also quantified separately at a commanded amplitude of 1000 mV.

**Figure 7:**
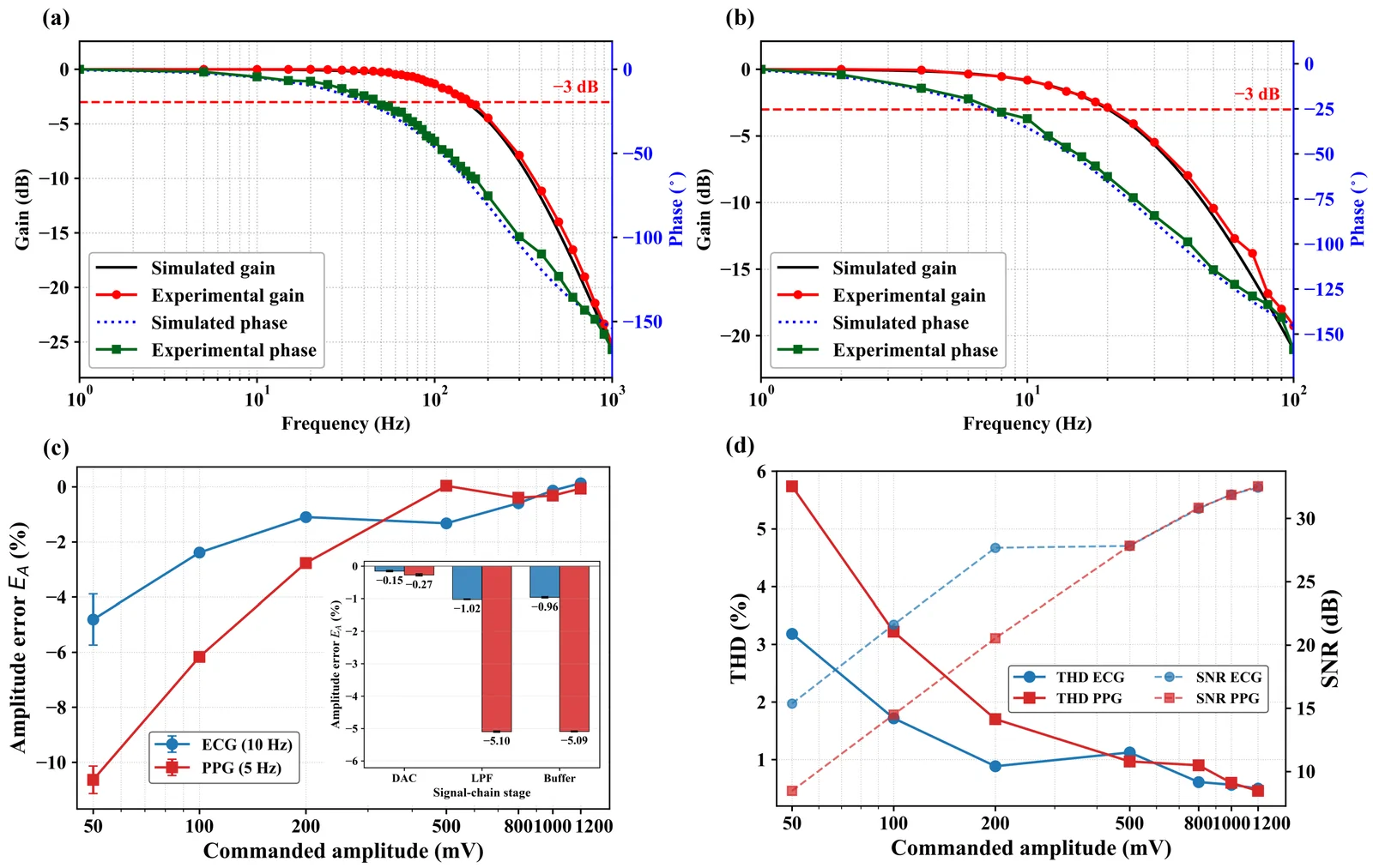
Characteristics of the analog circuit sequence: (**a**) amplitude and phase response of the ECG channel; (**b**) amplitude and phase response of the PPG channel; (**c**) amplitude error as a function of the set voltage, with the inset showing the error at the DAC, low-pass filter, and buffer at 1000 mV; and (**d**) THD and SNR as a function of the set voltage for both channels.

The measured bandwidths of both channels remained consistent with the design targets. The ECG channel exhibited a −3 dB cutoff frequency of 156.5 Hz compared with the design value of 150.7 Hz, corresponding to a deviation of 3.8%. The PPG channel reached 20.5 Hz compared with the target value of 20.1 Hz, giving a deviation of 2.2%. Simulated cutoff frequencies differed from the design values by less than 0.4% for both channels. The larger experimental deviations are consistent with passive-component tolerances. The cutoff frequency depends on the product RC, so the ±0.1% resistor tolerance contributes minimally, while the ±5% capacitor tolerance dominates the expected variation. Both measured deviations remain within this tolerance range. The ECG cutoff also remained above the 150 Hz upper frequency limit specified for diagnostic electrocardiography [25]. The measured phase responses followed the expected second-order behavior over the investigated frequency ranges (Figure 7a,b).

Amplitude accuracy improved with increasing signal level in both channels. The ECG error decreased from −4.81±0.93% at 50 mV to −0.14±0.03% at 1000 mV and 0.13±0.02% at 1200 mV. The PPG channel showed a similar trend, with errors of −10.64±0.50%, −0.32±0.03%, and −0.063±0.015% at the corresponding amplitudes. Across 800–1200 mV, the absolute error remained below 0.60% for both channels, an order of magnitude tighter than the ±5% or ±40μV deviation permitted by AAMI EC11 and IEC 60601-2-25. Stage-wise analysis at 1000 mV identified the LPF as the main source of amplitude attenuation. The DAC introduced errors of −0.15% for ECG and −0.27% for PPG, which increased to −1.02% and −5.10% after filtering. The buffer stage produced negligible additional change, with final errors of −0.96% and −5.09%, respectively.

Signal quality improved with increasing output amplitude, consistent with a predominantly amplitude-independent noise floor. In the ECG channel, THD decreased from 3.18±0.57% at 50 mV to 0.50±0.02% at 1200 mV, while SNR increased from 15.38±0.67 to 32.43±0.12 dB. The PPG channel showed a comparable trend, with THD decreasing from 5.74±0.34% to 0.46±0.01% and SNR increasing from 8.46±0.08 to 32.54±0.08 dB over the same range. The SNR increased by approximately 3.7 dB per doubling of output amplitude in the ECG channel and 5.3 dB in the PPG channel. Both values fall below the 6 dB expected of a strictly amplitude-independent noise floor, indicating that a component of the noise scales with signal level. The larger slope in the PPG channel is consistent with its lower filter cutoff, which attenuates a greater fraction of the broadband conversion noise. At commanded amplitudes of 800 mV and above, both channels maintained amplitude errors below 0.60%, THD below 1.00%, and SNR above 30 dB (Figure 7). This range was selected as the preferred operating region for calibration measurements.

#### 3.2.2. Timing, Synchronization, and Loop Integrity

**Figure 8:**
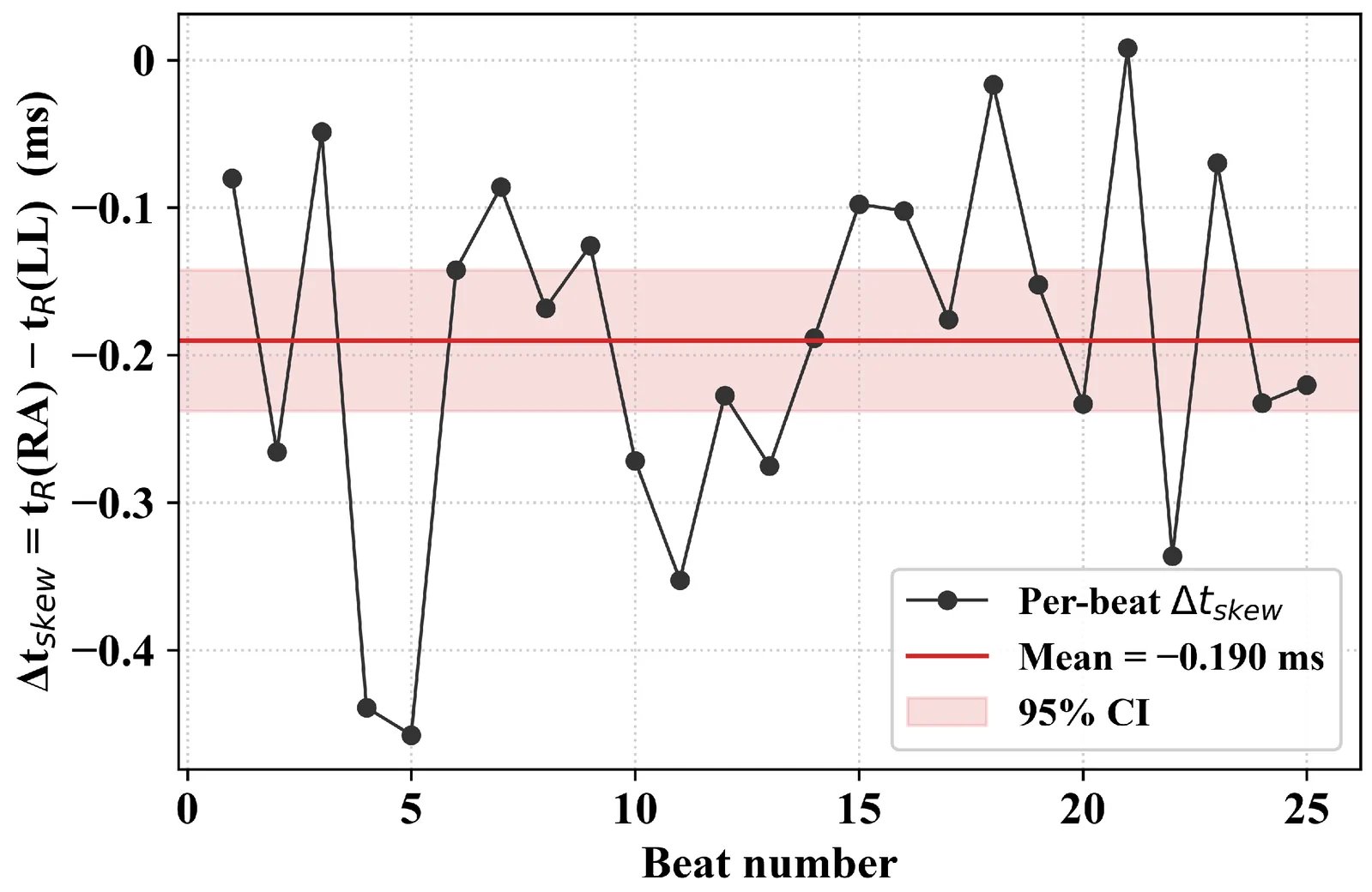
Inter-channel timing and playback stability of the ECG output system: beat-to-beat channel skew, $\Delta t_{\mathrm{skew}}$, between the LL and RA channels measured from common QRS complexes. Black dots connected by a line are the per-beat values; the red line and shaded band are the mean and its 95% confidence interval.

The timing synchronization relationship between channels was assessed by simultaneously recording the same QRS complex on the LL and RA outputs and comparing the R-peak times between the channels. Over 25 consecutive heartbeats, the mean deviation between the RA and LL channels was −0.19±0.12 ms, with a 95% confidence interval of [−0.24,−0.14] ms (Figure 8). The negative value indicates that the R-peak in the RA channel occurred an average of 0.19 ms earlier than the R-peak in the LL channel. The measured inter-channel skew was an order of magnitude smaller than the 2 ms sampling interval and approximately fifty times below the 10 ms time-axis misalignment permitted for multi-channel electrocardiographs by AAMI EC11. The corresponding timing error represented less than 0.1% of the pulse arrival times evaluated over the 200–290 ms range. A skew below one sampling interval is required for the prescribed electromechanical delay to be reproduced without channel-dependent bias, so that the measured pulse arrival time reflects the coupling model rather than the output stage.

**Figure 9:**
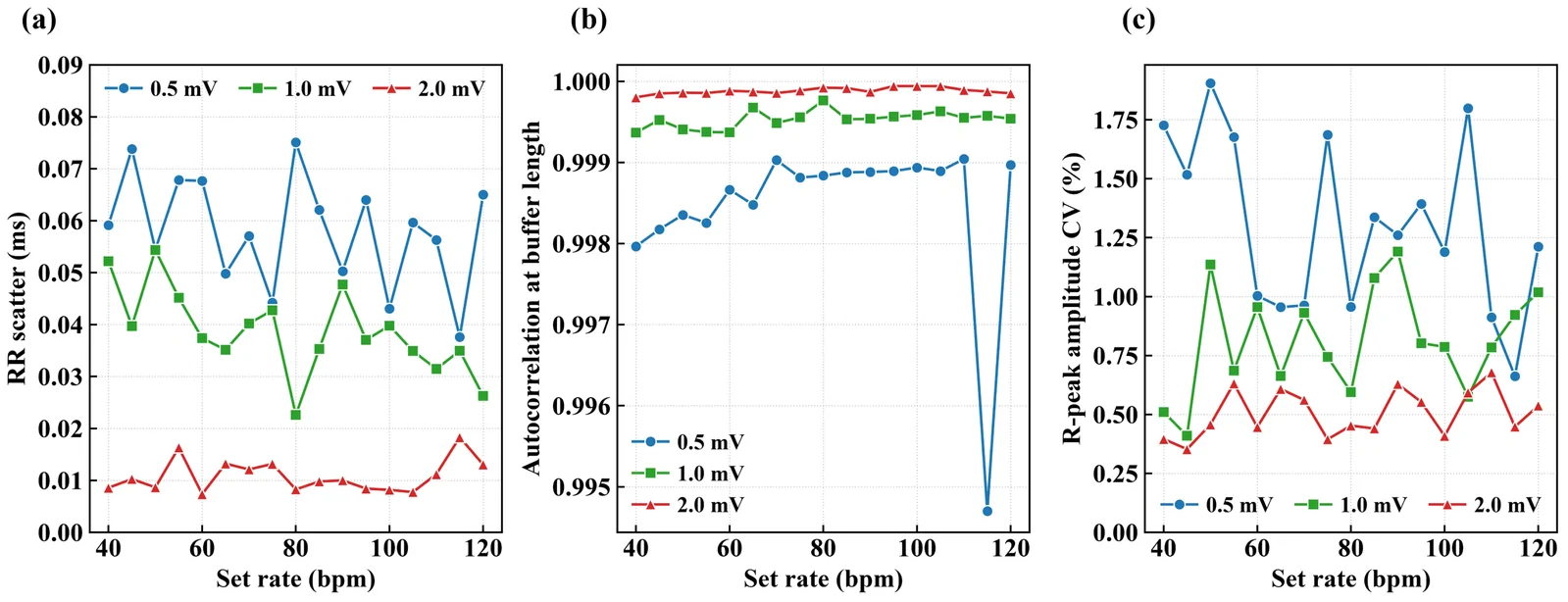
Playback stability of the ECG output across set heart rates: (**a**) RR-interval scatter, (**b**) waveform autocorrelation at the playback-buffer length, and (**c**) R-peak amplitude coefficient of variation, each at set rates of 40–120 bpm and three signal sensitivities (0.5, 1.0, 2.0 mV).

The playback stability of the system was evaluated across the programmed heart-rate range and output sensitivity levels using three metrics, including RR-interval dispersion within each recording, the autocorrelation coefficient at the playback-buffer length, and the coefficient of variation of the R-peak amplitude. These metrics were examined over heart rates ranging from 40 to 120 bpm at three sensitivity levels of 0.5, 1.0, and 2.0 mV, as shown in Figure 9.

The mean RR-interval scatter was 0.058, 0.039, and 0.011 ms at sensitivity settings of 0.5, 1.0, and 2.0 mV, respectively, with a maximum value below 0.075 ms (Figure 9a). This value was approximately 25 times smaller than the 2 ms sampling period, indicating low temporal variation between consecutive playback cycles. The autocorrelation coefficient ranged from 0.9985 to 0.9999, with a minimum value of 0.995 at 0.5 mV. These values indicate consistent waveform repetition without appreciable morphological discontinuity during buffer cycling (Figure 9b). The mean coefficient of variation of the R-peak amplitude decreased from 1.30% at 0.5 mV to 0.81% at 1.0 mV and 0.51% at 2.0 mV (Figure 9c). All three metrics improved as the sensitivity increased, consistent with the higher SNR at larger signal amplitudes. The system maintained timing stability, playback-loop continuity, and amplitude repeatability across the investigated heart-rate range, with no evident degradation as the set heart rate increased.

#### 3.2.3. Output Stability

**Figure 10:**
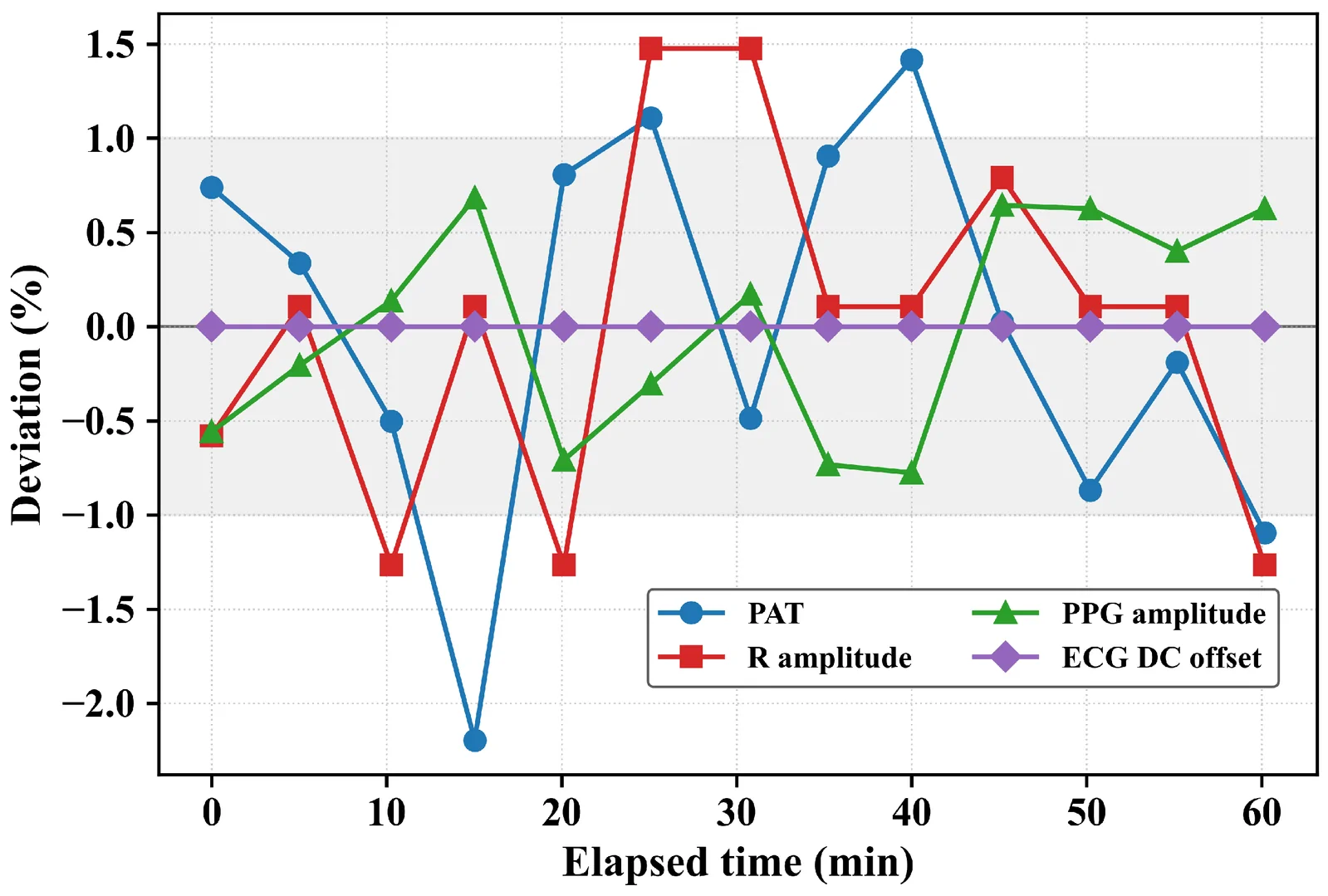
Output stability during 60 min of continuous operation. Deviations in PAT, R-peak amplitude, PPG amplitude, and ECG DC offset from their respective session means were evaluated at 5-min intervals. No significant drift was observed in any metric, and the shaded region indicates the ±1% range.

Long-term output stability was assessed over 60 min of continuous operation by recording measurements at 5 min intervals. PAT, R-peak amplitude, PPG pulse amplitude, and ECG DC offset were expressed as deviations from their respective session means (Figure 10). The drift rates of PAT, ECG amplitude, and PPG amplitude were −0.49%/h, +0.33%/h, and +0.75%/h, respectively, while the ECG DC offset remained approximately constant. The largest of these rates is more than an order of magnitude below the 0.33%/min gain-change rate permitted for diagnostic ECG by AAMI EC11, and the total variation over the hour remained within ±1.5% of the session mean, half the ±3% total change the same standard allows over an equivalent interval. Both amplitude metrics remained within the shaded ±1% range. Timing, amplitude, and baseline level therefore remained within diagnostic-recorder tolerances over 60 min of continuous operation, matching the gain-stability interval specified by the standard.

#### 3.2.4. Clinical-Record Playback and End-to-End Fidelity

**Figure 11:**
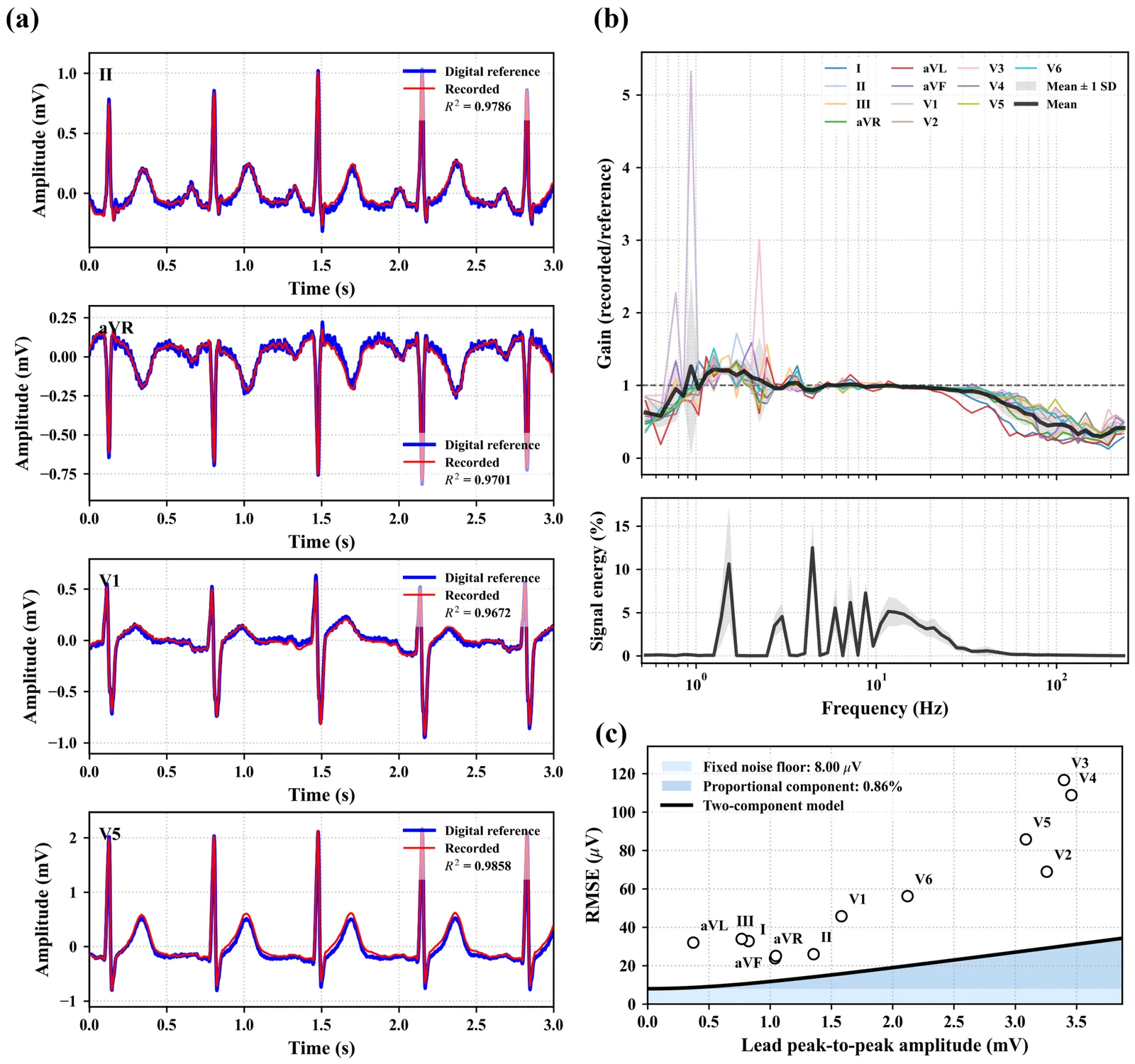
End-to-end reproduction of a clinical 12-lead recording: (**a**) the digital reference and recorded waveforms for four representative leads, namely II, aVR, V1, and V5; (**b**) the transfer gain across all twelve leads together with the corresponding frequency distribution of reference-signal energy; and (**c**) the decomposition of the reproduction error into a fixed noise floor and an amplitude-proportional component.

A 10 s clinical 12-lead ECG recording sampled at 500 Hz and obtained from the PhysioNet database was used to evaluate end-to-end reconstruction fidelity [33]. The playback buffer contained 4736 samples, corresponding to a duration of 9.47 s and covering 94.7% of the original recording. The maximum electrode potential was 2.75 mV. Figure 11 presents the waveform similarity in representative leads, the frequency-domain transmission characteristics, and the interlead distribution of reconstruction errors.

After phase alignment, leads II, aVR, V1, and V5 achieved $R^{2}$ values ranging from 0.967 to 0.986. The cycle-to-cycle correlation coefficient between successive buffer repetitions reached 0.99618, confirming stable and repeatable waveform playback (Figure 11a). Across all 12 leads, the mean regression slope increased from 0.968±0.038 under broadband evaluation to 0.997±0.016 within the 0.5–40 Hz band. Over the same frequency range, the mean $R^{2}$ increased from 0.953±0.051 to 0.973±0.034, whereas the PRD decreased from 27.55±13.49% to 14.82±8.66%. The mean gain remained close to unity within 5–20 Hz, decreased to 0.949 within 20–40 Hz, and exhibited greater attenuation above 40 Hz. The analysis showed that 76.4% of the reference-signal energy was concentrated within 0.5–40.0 Hz, indicating that out-of-band discrepancies contributed substantially to the broadband reconstruction error (Figure 11b). The RMSE generally increased with lead amplitude but exceeded the values predicted by the nominal model comprising an 8.0 μV noise floor and a 0.86% proportional component, particularly in leads V2–V5. Unconstrained fitting yielded a noise floor of 17.84±9.37μV and a proportional component of 2.75±0.21%, indicating that the actual error contained lead-dependent components that were not fully described by the nominal two-component model (Figure 11c). The estimated noise floor was comparable in magnitude to the 30 μV peak-to-peak internal noise limit specified by AAMI EC11 for diagnostic ECG. Direct comparison remains limited by the different definitions, as the fitted value represents an RMS intercept of reconstruction error across leads, whereas the standard specifies peak-to-peak noise measured with shorted recorder inputs. The emulator reproduction error was consequently of the same order as the allowable internal noise of the recording device under evaluation. Some low-amplitude leads, including aVL and III, exhibited relatively high PRD values. Therefore, PRD should be interpreted together with absolute RMSE rather than used as an independent fidelity metric. The buffer length was set by the microcontroller SRAM available for waveform storage, since ten 16-bit channels at this depth occupy approximately 94.7 kB. Playback stability was assessed from continuous repetition of the buffered segment rather than from a single long record, with no cumulative drift over 60 min of operation. Longer recordings were not evaluated, and reproducing rhythm variability on an ambulatory scale would require streaming playback or external memory.

### 3.3. Response of a Commercial Electrocardiograph

#### 3.3.1. Rate and Amplitude Acceptance

**Figure 12:**
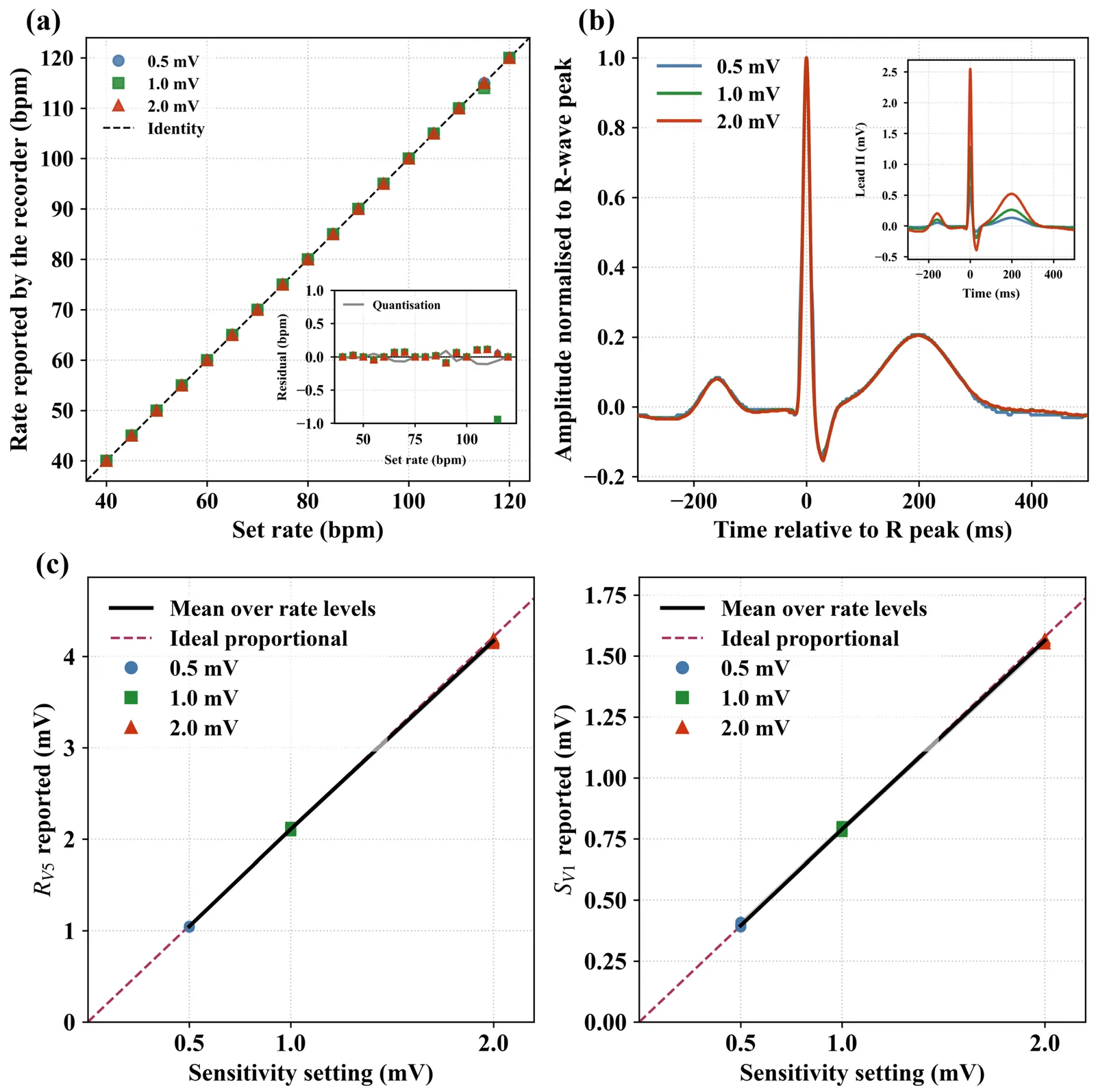
Verification of the emitted ECG signal using the Kenz Cardico 306 electrocardiograph: (**a**) recorder-reported heart rate versus the programmed rate from 40 to 120 bpm at sensitivity settings of 0.5, 1.0, and 2.0 mV, with the identity line indicating ideal agreement, while the inset shows the residual relative to the realizable rate imposed by integer-length buffer quantization; (**b**) representative lead II beats at the three sensitivity settings after normalization to the R-wave peak, showing preservation of waveform morphology, with the inset presenting the corresponding waveforms before normalization on the absolute amplitude scale; and (**c**) reported $R_{V5}$ and $S_{V1}$ amplitudes as functions of the sensitivity setting, where thin gray lines represent individual rate levels, the thick black line represents the mean across rates, and the dashed line indicates the ideal proportional response through the origin.

A commercial electrocardiograph was used to verify its ability to acquire the transmitted signals. Normal sinus rhythm was evaluated over the range of 40–120 bpm in 5 bpm increments at three amplitude levels of 0.5, 1.0, and 2.0 mV. Figure 12 presents the heart-rate accuracy reported by the Kenz Cardico 306, the preservation of waveform morphology after amplitude normalization, and the linear responses of $R_{V5}$ and $S_{V1}$.

The recorder returned the programmed heart rate in 50 of the 51 test conditions. The only discrepancy occurred at 115 bpm and 1.0 mV, for which the reported heart rate was 114 bpm (Figure 12a). The mean absolute error relative to the programmed heart rate was 0.02 bpm, whereas the difference between the programmed and achievable heart rates resulting from the finite buffer length did not exceed 0.110 bpm. This value was lower than the 1 bpm display resolution of the recorder. After normalization to the R-wave peak, the lead II waveforms at the three amplitude levels achieved a minimum correlation coefficient of 0.9964 and RMS deviations ranging from 0.28% to 1.19%, indicating that amplitude variation did not substantially alter the transmitted waveform morphology (Figure 12b). The maximum deviations occurred within ±11 ms of the R-wave peak and corresponded to temporal offsets of 0.4–2.0 ms, which were comparable to the 2 ms sampling interval of the recorder. The negative correlation between sampling phase and amplitude deviation (r=−0.607), together with an R-wave peak-amplitude coefficient of variation of only 0.70%, indicated that the local discrepancies were primarily associated with asynchronous sampling between the signal generator and the recorder rather than with actual waveform variation. The amplitude response also remained close to ideal proportionality. The 1.0/0.5, 2.0/0.5, and 2.0/1.0 ratios of $R_{V5}$ and $S_{V1}$ deviated by less than 1.05%, with a minimum $R^{2}$ of 0.999 across the entire heart-rate range (Figure 12c). These results indicate that the transmitted signals maintained amplitude linearity and morphological invariance. Therefore, the variation in QRS duration reported by the Cardio Ken across amplitude levels was more consistent with errors in onset and offset detection by the recorder algorithm than with changes in the input signal.

#### 3.3.2. Automated Interval Bias and Measurement Limits

The preceding waveform analysis showed that changes in input amplitude did not materially alter the normalized waveform morphology. Therefore, the interval variations reported at 0.5, 1.0, and 2.0 mV primarily reflected the amplitude-dependent response of the Kenz Cardico 306 delineation algorithm rather than programmed changes in the input waveform.

**Figure 13:**
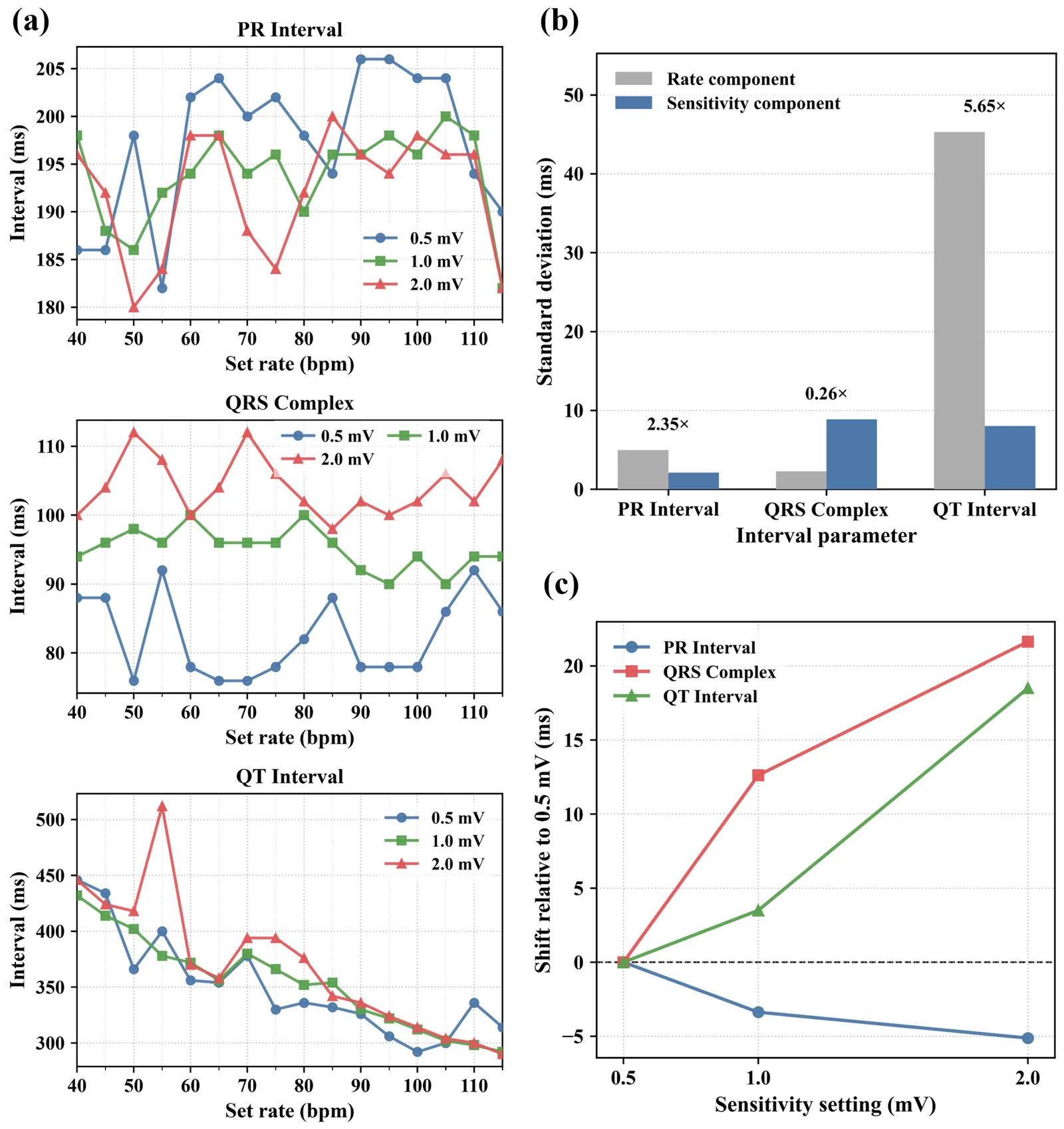
Amplitude dependence of the intervals reported by the commercial electrocardiograph: (**a**) recorder-reported PR, QRS, and QT intervals across heart rates and sensitivity settings; (**b**) standard-deviation components associated with heart rate and sensitivity setting; and (**c**) mean interval shifts relative to the 0.5 mV setting.

After excluding the 120 bpm condition, the reported PR interval decreased from 197.25±7.76 ms at 0.5 mV to 193.88±5.03 ms at 1.0 mV and 192.12±6.47 ms at 2.0 mV. The corresponding paired mean shifts relative to 0.5 mV were −3.38 and −5.13 ms. In contrast, QRS duration increased from 82.50±5.91 to 95.12±2.92 and 104.12±4.22 ms, corresponding to shifts of 12.63 and 21.63 ms (Figure 13a). These opposite trends are consistent with an amplitude-dependent displacement of the detected QRS onset, although the unavailable internal algorithm prevents direct confirmation of this mechanism. The QT interval increased from 350.38±45.20 ms at 0.5 mV to 368.88±60.73 ms at 2.0 mV (Table 3). The greater variability of the QT interval across heart rate levels suggests a stronger dependence on heart rate and T-wave delineation.

Variance decomposition confirmed that QRS duration was the interval most affected by input amplitude (Figure 13b). Its sensitivity-related SD was 8.87 ms, approximately 3.90 times the rate-related SD of 2.27 ms. In comparison, PR and QT were dominated by rate-related variation, with rate- and sensitivity-related SDs of 4.99 and 2.13 ms for PR and 45.30 and 8.02 ms for QT, respectively. The low rate-dependent $R^{2}$ values for PR, ranging from 0.019 to 0.161, indicate weak associations with heart rate. The paired shifts relative to 0.5 mV are summarized in Figure 13c, where PR and QRS changed in opposite directions, while QT showed a more variable positive shift. The 120 bpm condition was excluded from the pooled analysis according to the predefined validity criterion because the recorder did not return a valid PR interval and produced an abrupt QT increase. The recorder accepted the generated signals across the complete amplitude range, but its reported intervals were not invariant to input amplitude. The emulator can therefore support both reproducible signal calibration and the identification of amplitude-dependent bias in automated ECG measurement algorithms.

#### 3.3.3. Demonstration of Interpretation-Algorithm Stress Testing

**Figure 14:**
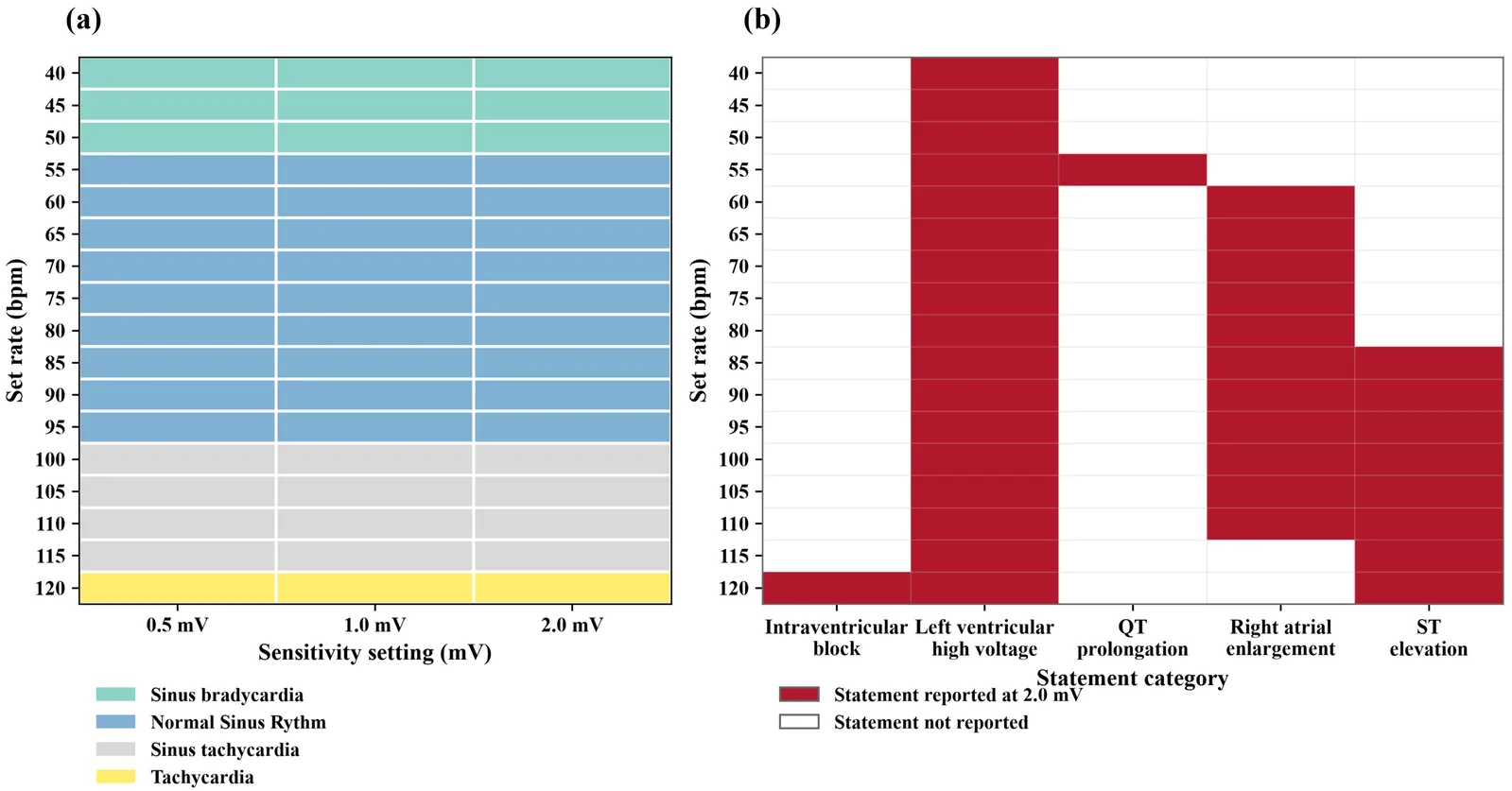
Characterization of the electrocardiograph interpretation algorithm with a known input: (**a**) rhythm label as a function of the emitted rate and amplitude setting; and (**b**) diagnostic statements detected only at the highest amplitude setting, where $R_{V5}$ reaches 4.17 mV and exceeds the voltage criterion for left ventricular hypertrophy. Red cells indicate that a statement was reported, whereas white cells indicate that it was not; colours represent presence/absence only.

A tool for examining the electrocardiograph’s interpretation algorithm was used to demonstrate the applicability of the signal generator. The diagnostic labels reported by the Kenz Cardico 306 were summarized according to the programmed heart rate and signal sensitivity setting. Figure 14a presents the rhythm labels over the range of 40–120 bpm at amplitude levels of 0.5, 1.0, and 2.0 mV, whereas Figure 14b summarizes the diagnostic statements observed only at 2.0 mV.

The results showed that the baseline rhythm labels were completely consistent across the three amplitude levels, with Kenz Cardico 306 classifying 40–50 bpm as Sinus bradycardia, 55–95 bpm as Normal Sinus Rhythm, 100–115 bpm as Sinus tachycardia, and 120 bpm as Tachycardia (Figure 14a). Since the input rhythm was pre-set and accurately validated in Section 3.3.1, the label transition points can be considered the effective thresholds of the algorithm under the survey conditions. With a 5 bpm scan step, the boundary between sinus bradycardia and normal sinus rhythm is in the range of 50–55 bpm, while the boundary between normal sinus rhythm and sinus tachycardia is in the range of 95–100 bpm. The appearance of the label Tachycardia at 120 bpm indicates that the algorithm uses this general term at a rhythm level higher than the threshold of the label Sinus tachycardia.

The diagnostic statements reported below were obtained through controlled amplitude scaling of synthetic waveforms. The resulting classifications reflect the decision boundaries of the recorder interpretation algorithm rather than clinical observations. No patient data, pathological condition, or physiological substrate contributed to these findings. Figure 14b summarizes the corresponding amplitude-dependent interpretation response of the Kenz Cardico 306. Five diagnostic statements appeared only at 2.0 mV, including left ventricular high voltage (n=17), right atrial enlargement (n=11), ST elevation (n=8), QT prolongation (n=1), and intraventricular block (n=1). Among these statements, left ventricular high voltage occurred across the entire range of 40–120 bpm, consistent with the increase in $R_{V5}$ from 2.11 mV at the 1.0 mV setting to 4.17 mV at the 2.0 mV setting. The occurrence of right atrial enlargement and ST elevation indicates that global amplitude scaling affected not only the QRS complex but also the P wave and ST segment, thereby activating multiple interpretation criteria.

## 4. Discussion

The proposed framework generates ECG and PPG signals from a shared atrial and ventricular event sequence. Separation of pacing, conduction, and ventricular activation supports Mobitz type I, complete atrioventricular dissociation, dropped beats, PR prolongation, and independent ventricular rhythms within a unified model. Agreement between conduction parameters indicates that signal timing is governed by the event schedule. The same ventricular sequence drives PPG generation, allowing ectopic beats to produce delayed and attenuated peripheral pulses without post-generation alignment. The modular organization also facilitates extension to additional rhythms by modifying the corresponding pacing or conduction rules. Mobitz type II can be modeled by fixed-ratio block instead of progressive PR prolongation. Premature ventricular contractions are introduced by adding ectopic ventricular events that yield wide QRS complexes and delayed, attenuated PPG responses.

The shared event structure differs from playback-based simulators, which remain constrained by the rhythms present in recorded data [13,15], and from model-based ECG generators without an explicitly coupled peripheral channel [10,16]. Multimodal simulators that combine a cardiac signal with a peripheral pulse have been reported, but they reproduce previously recorded signal pairs and cannot generate coupling relationships absent from the source data [20]. Across the systems summarized in Table 4, no prior approach derives a 12-lead ECG and a peripheral pulse from a common activation sequence. The proposed framework addresses this gap by generating both signals from a shared conduction schedule. Blocked atrial events consequently produce no peripheral pulse, whereas ectopic ventricular events produce attenuated pulse responses without independent case-specific specification. Clinical ECG recordings also enter the same electrode-domain pathway and were reproduced with correlation coefficients of 0.967–0.986.

The measured PAT error in sinus rhythm was 8.39 ms, close to the predicted differential filter delay of 8.84 ms. The agreement indicated that the dominant timing offset originates from the analog filtering stage rather than from the electromechanical model. The deterministic delay can be compensated in firmware by advancing the PPG stream by approximately four to five samples at a 500 Hz update rate. Ventricular tachycardia produced a larger PAT error of 29.83 ms. The additional residual of approximately 21 ms is consistent with reduced pulse-foot detection accuracy at low PPG amplitudes.

The study still has several limitations. ECG outputs were validated using a commercial electrocardiograph, whereas PPG evaluation was limited to analog voltage measurements. Commercial pulse oximeters respond to modulated light reaching a photodiode rather than to an applied voltage, so validation against optical devices would require an additional light-emitting stage that the present hardware does not include. Respiratory modulation and baseline drift were disabled during evaluation to provide deterministic reference signals, limiting assessment of drift rejection and low-SNR detection. Both features remain configurable within the model. Clinical playback was limited to 9.47 s, and PPG amplitude error increased to approximately −10.6% at 50 mV. The event-based formulation does not represent fibrillation, dynamic ischemia, concealed conduction, or high-frequency QRS features. The 500 Hz update rate also limits higher-bandwidth applications. The demonstrated performance supports the use of the system as a deterministic and synchronized 12-lead ECG–PPG source for controlled evaluation of acquisition hardware and signal-processing algorithms. Further development should address the identified limitations by improving low-amplitude accuracy, incorporating an optical PPG output stage, and extending validation to conditions with controlled modulation, baseline drift, and noise.

## 5. Conclusions

This study presents a programmable ECG–PPG physiological signal simulator, combining an event-driven mathematical model with a synchronized multichannel analog hardware platform. The model represents atrial rhythm, atrioventricular conduction, and ventricular activity as separate blocks and infers peripheral impulses from ventricular events via electromechanical delay. The system reconstructs a range of normal and abnormal heart rhythms along with their corresponding hemodynamic responses and can replay clinical 12-lead recordings via the same path. Verification against a commercial electrocardiograph and at the analog output confirmed accurate conduction timing, high amplitude accuracy, morphological fidelity in the medium-to-high output range, and long-term stable operation, while also indicating the low-amplitude and probe-dependent limitations to which these claims apply. In addition to calibration, deterministic and repeatable signals enable controlled robustness testing of interval measurement algorithms and recorder interpretation. This design is low-cost and open-source, supporting device quality control, medical training, and physiological signal processing research. Further studies will focus on improving the accuracy of low-amplitude signals, integrating breath and baseline correction capabilities, and extending the technological framework to generate labeled datasets for data-driven ECG and PPG analysis.

## Figures and Tables

**Table 1:** Conduction parameters, prescribed and measured.

Rhythm ^1^	Parameter	Set	Measured (Cardio Ken)	Error
NSR	PR	160 ms	161.23 ± 0.58 ms	1.23 ms
1° AVB	PR	240 ms	241.66 ± 0.16 ms	1.66 ms
Mobitz I	PR range/group	160–240 ms	161.13 ± 0.32–241.16 ± 0.25 ms	1.13/1.16 ms
Mobitz I	ΔPR per beat	40 ms	40.02 ± 0.04 ms	0.02 ms
CAVB	Atrial/vent. rate	75/38 bpm	76.06 ± 0.07/38.02 ± 0.00 bpm	1.06/0.02 bpm
AT	Atrial rate/QRS	160 bpm/<120 ms	159.59 ± 0.00 bpm/87.40 ± 3.33 ms	−0.41 bpm
VT	Rate/QRS width	160 bpm/≥120 ms	159.57 ± 0.00 bpm/234.63 ± 5.75 ms	−0.43 bpm

^1^ NSR—normal sinus rhythm, 1° AVB— first-degree atrioventricular block, Mobitz I—second-degree atrioventricular block type I (Wenckebach), CAVB—complete atrioventricular block, AT—atrial tachycardia, and VT—ventricular tachycardia.

**Table 2:** PAT preservation through the signal chain.

Beat Class	PAT Set (ms)	PAT Analog (ms)	$E_{\mathrm{PAT}}$ (ms)	Amplitude Ratio
Sinus rhythm	209.09	217.48	8.39	1.00
Ventricular	244.09	273.92	29.83	0.44

**Table 3:** Intervals reported at three sensitivity settings.

Sensitivity	PR (ms) ^1^	QRS (ms) ^1^	QT (ms) ^1^
0.5 mV	197.25±7.76	82.50±5.91	350.38±45.20
1.0 mV	193.88±5.03	95.12±2.92	353.88±42.24
2.0 mV	192.12±6.47	104.12±4.22	368.88±60.73
Shift	−5.13	+21.63	+18.50

^1^ The analysis included 16 rate levels from 40 to 115 bpm. Shifts were calculated relative to the paired measurements at 0.5 mV. The 120-bpm condition was excluded according to the predefined interval-validity criterion.

**Table 4:** Comparison of representative physiological signal simulators. Entries marked “NR” were not reported in the corresponding publication.

System	Signals	Approach	Synchronous Channels	AV Block	Beat-Class Coupling	Timing Accuracy	Hardware	Validation Grid (Rates × Amps)
Patil et al. [15]	ECG	Playback	9	No	—	NR	12-bit/NR	NR
Das et al. [13]	ECG	Playback	1 (9 multiplexed)	No	—	NR	8-bit/NR	2 rates × 1 amplitude
Edelmann et al. [10]	ECG	Model/hybrid	NR	No	—	NR	16-bit/NR	NR
Quiroz et al. [16]	ECG	Model	12 (derived)	Yes	—	NR	12-bit/NR	NR
Delgerkhaan et al. [20]	PCG + PPG	Playback	2	—	No	NR	32&12-bit/44.1 kHz	1 amplitude
This work	ECG + PPG	Event-driven + playback	10	Yes	Yes	<2 ms skew	12-bit/500 Hz	51 conditions (17 rates × 3 amps)

## Data Availability

The data and resources supporting the findings of this study are openly available in the ECG_PPG_Simulation repository (version 1.0.0) on GitHub at https://github.com/VHT-designer/ECG_PPG_Simulation (accessed on 19 July 2026). The repository includes the PC graphical user interface (GUI), the STM32F407VET6 firmware, and the hardware schematics and Gerber files for the printed circuit board.

## Abbreviations

The following abbreviations are used in this manuscript:

CV	Coefficient of Variation
DAC	Digital-to-Analog Converter
ECG	Electrocardiography
ECGSYN	Electrocardiogram Synthesizer
LPF	Low-Pass Filter
PAT	Pulse Arrival Time
PEP	Pre-Ejection Period
PPG	Photoplethysmography
PRD	Percentage Root-Mean-Square Difference
RMSE	Root Mean Square Error
SNR	Signal-to-Noise Ratio
SPI	Serial Peripheral Interface
THD	Total Harmonic Distortion
UART	Universal Asynchronous Receiver–Transmitter
WCT	Wilson Central Terminal
WHO	World Health Organization
